# Integrative “Omics”-Approach Discovers Dynamic and Regulatory Features of Bacterial Stress Responses

**DOI:** 10.1371/journal.pgen.1003576

**Published:** 2013-06-20

**Authors:** Bork A. Berghoff, Anne Konzer, Nils N. Mank, Mario Looso, Tom Rische, Konrad U. Förstner, Marcus Krüger, Gabriele Klug

**Affiliations:** 1 Institut für Mikrobiologie und Molekularbiologie, Justus-Liebig-Universität, Giessen, Germany; 2 Department of Cardiac Development and Remodelling, Max-Planck-Institute for Heart and Lung Research, Bad Nauheim, Germany; 3 Institute for Molecular Infection Biology, University of Würzburg, Würzburg, Germany; 4 Research Center for Infectious Diseases, University of Würzburg, Würzburg, Germany; Université Paris Descartes, INSERM U1001, France

## Abstract

Bacteria constantly face stress conditions and therefore mount specific responses to ensure adaptation and survival. Stress responses were believed to be predominantly regulated at the transcriptional level. In the phototrophic bacterium *Rhodobacter sphaeroides* the response to singlet oxygen is initiated by alternative sigma factors. Further adaptive mechanisms include post-transcriptional and post-translational events, which have to be considered to gain a deeper understanding of how sophisticated regulation networks operate. To address this issue, we integrated three layers of regulation: (1) total mRNA levels at different time-points revealed dynamics of the transcriptome, (2) mRNAs in polysome fractions reported on translational regulation (translatome), and (3) SILAC-based mass spectrometry was used to quantify protein abundances (proteome). The singlet oxygen stress response exhibited highly dynamic features regarding short-term effects and late adaptation, which could in part be assigned to the sigma factors RpoE and RpoH2 generating distinct expression kinetics of corresponding regulons. The occurrence of polar expression patterns of genes within stress-inducible operons pointed to an alternative of dynamic fine-tuning upon stress. In addition to transcriptional activation, we observed significant induction of genes at the post-transcriptional level (translatome), which identified new putative regulators and assigned genes of quorum sensing to the singlet oxygen stress response. Intriguingly, the SILAC approach explored the stress-dependent decline of photosynthetic proteins, but also identified 19 new open reading frames, which were partly validated by RNA-seq. We propose that comparative approaches as presented here will help to create multi-layered expression maps on the system level (“expressome”). Finally, intense mass spectrometry combined with RNA-seq might be the future tool of choice to re-annotate genomes in various organisms and will help to understand how they adapt to alternating conditions.

## Author Summary

Bacteria are frequently exposed to disadvantageous conditions, like elevated temperatures or nutrient depletion. The ability to maintain viable populations is based on cellular stress responses, which are regulated in a complex manner with different outputs on different regulatory levels. For example, mRNA levels do not ultimately determine protein amounts since translation of mRNAs can be influenced irrespective of mRNA levels. To appreciate nature and frequency of these regulatory events, multi-layered experimental approaches are required on a global scale. The photo-oxidative stress response of the purple bacterium *Rhodobacter sphaeroides* was chosen as a model. Changes of total mRNAs (transcriptome) and ribosomal-bound mRNAs (translatome) were monitored by microarrays. The proteome was assessed by mass spectrometry, applying a “bacterial SILAC standard” for indirect quantification, an approach which additionally identified new open reading frames. Integration of the three expression levels provided a comprehensive insight into regulatory events and identified new stress-responsive genes, including genes for transcriptional regulators and for quorum sensing. We found that translational control exceeds simple regulation on the transcriptional level. Furthermore, polar expression patterns within inducible operons point at the possibility of expression fine-tuning by gene positioning.

## Introduction

All living organisms constantly remodel mRNA and protein abundances as a response to environmental factors or in the course of development and differentiation. In order to realize adequate responses, gene expression has to be controlled by sophisticated regulation networks. Besides transcriptional regulation, it is now broadly appreciated that post-transcriptional and post-translational events have a non-negligible importance and help to explain the discrepancy between mRNA and protein levels regularly observed in biological systems [1].

Interestingly, mRNA-protein correlations might be fairly high, with Pearson coefficients ranging between 0.66 and 0.76 as measured for the budding yeast *Saccharomyces cerevisiae* [2], [3]. Weaker correlations are assumed to be partly biased by methodological constraints, and technical improvements therefore tend to increase the measured correlations [4]. However, a significant portion of all genes are obviously subject to post-transcriptional regulation, as demonstrated for one-third of all genes in *Saccharomyces cerevisiae*, exhibiting an altered translational efficiency upon starvation [5]. In the genome-reduced bacterium *Mycoplasma pneumoniae* it was recently shown that translational control has a stronger regulatory influence on protein levels than protein turnover [6], which clearly underlines the importance of assessing the translatome for gene regulation studies. The translatome is defined as the sum of mRNAs captured in ribosomes for translation. Several studies employed ribosome profiling combined with microarray-based methods to calculate changes in actively translated mRNAs. For example, in the haloarchaeal model species *Halobacterium salinarum* and *Haloferax volcanii* translational efficiency was monitored at different growth stages [7], while in the gram-positive bacterium *Lactococcus lactis* and in the fission yeast *Schizosaccharomyces pombe* ribosome occupancy and ribosome density were assigned [8], [9]. Other attempts included ribosome footprinting together with deep sequencing of RNA or purification of affinity-tagged ribosomes followed by microarray analysis in *Saccharomyces cerevisiae* [5], [10]. However, none of these studies directly compared translatomic data to both transcriptomic and proteomic data.

Quantitative proteomics is still the bottleneck of comparative approaches, since the numbers of identified proteins regularly drag behind expected numbers. In yeast extensive mass spectrometry (MS)-based proteomics was applied to overcome this problem and coverage of the entire proteome was finally claimed by identification of ∼4.400 proteins [11], and recently, a stunning number of nearly 12.000 proteins were identified in human cell lines [12]. However, these might be outstanding cases, even though advances in MS-based proteomics are steadily increasing. One of the main tasks is the accurate quantification of changes in protein abundance between different cellular states. The SILAC method (stable isotope labeling of amino acids in cell culture) addresses this problem by the use of heavy amino acids [13]. Peptides either contain the heavy or the light form of amino acids – usually arginine and lysine – and therefore give a distinct mass difference which enables quantification by direct comparison of peptide peak intensities. SILAC is the method of choice for mammalian systems, but has also been applied to newts, nematodes, yeast, and bacteria like *Escherichia coli* and *Bacillus subtilis* [11], [14]–[19]. However, in bacteria SILAC-based proteomics and the use of translatomics as described above are highly under-represented when compared to transcriptome experiments.

In order to gain a comprehensive picture of bacterial regulation, several “omics” should be applied simultaneously in an integrative approach. To follow up such a strategy, *Rhodobacter sphaeroides* was chosen as model for the investigation of bacterial stress responses. *Rhodobacter* species are well investigated with regard to regulation of photosynthesis genes [20]–[22], and in particular *R. sphaeroides* has been established for studying the photo-oxidative stress response in anoxygenic phototrophs [23], [24]. Photo-oxidative stress occurs whenever singlet oxygen is generated, which mainly happens during photosynthesis [25]. We systematically investigated the transcriptome at early and late time-points of the stress response by microarray analysis, which revealed expression dynamics for stress-dependent mRNAs but also small regulatory RNAs (sRNAs). In addition, stress-specific sigma factor regulons were analyzed. The proteome was assessed by SILAC-based MS, using an indirect quantification approach by applying a heavy standard consisting of different culture conditions. Finally, changes of mRNAs in polysome fractions (translatome) were measured by microarray analysis to investigate translational regulation, which closed the gap between mRNA and protein levels. This is one of the most comprehensive studies on bacterial stress responses reported so far, combining several “omics” for genome-wide applications, and will serve as an example for future perspectives in bacterial system biology.

## Results

**Figure 1:**
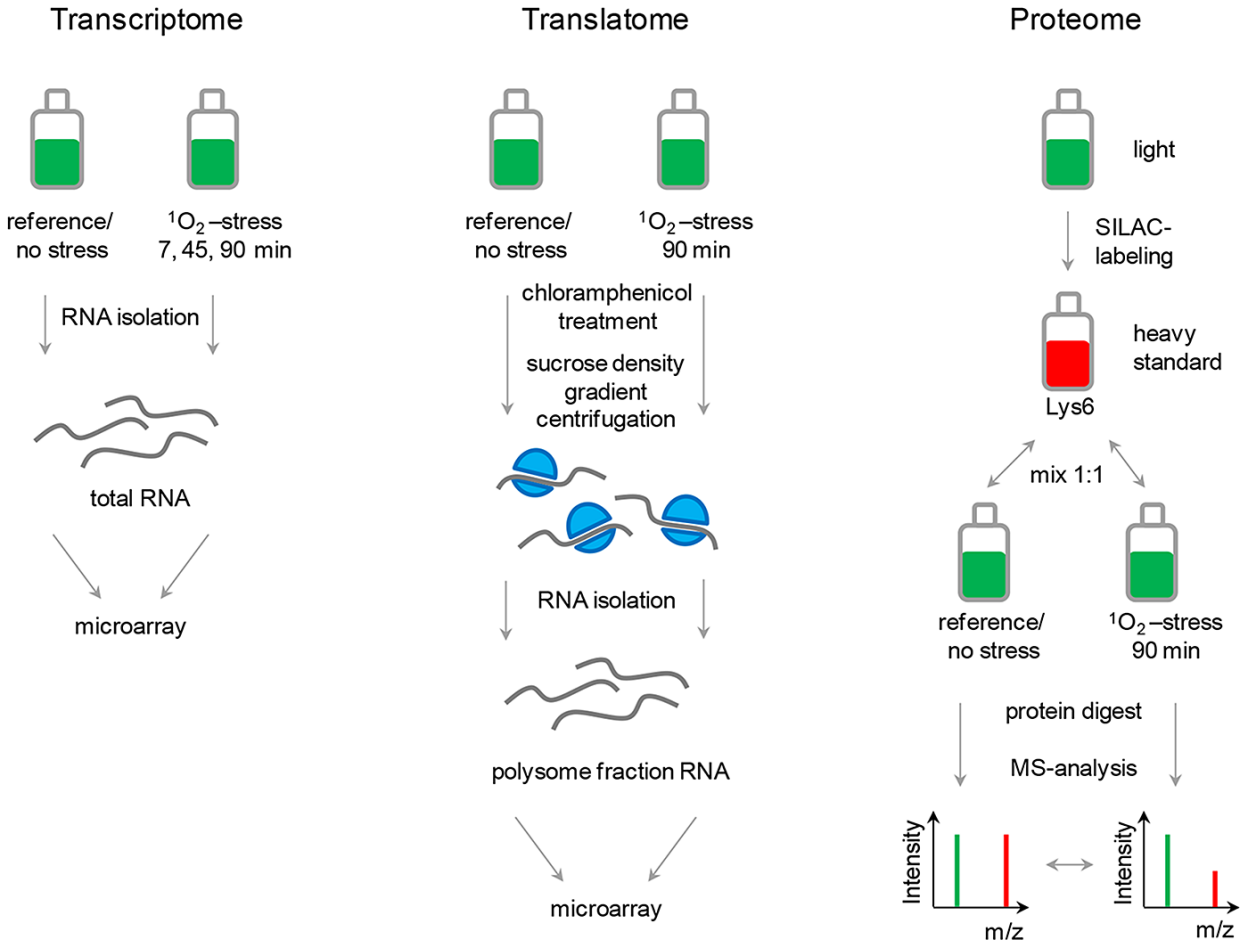
Design of a comparative “omics”-approach for bacterial stress responses. Bacterial cultures are subjected to stress (e.g., singlet oxygen, ^1^O_2_) and relative changes monitored for mRNAs in total RNA isolates (transcriptome), mRNAs in polysomes (translatome), and for proteins (proteome). For the proteome approach, a culture has to be fully labeled with the heavy amino acid ^13^C_6_-lysine (Lys6; heavy standard). Protein samples from a reference (no stress) or stressed cells are separately mixed in a 1∶1 ratio with heavy standard protein and subsequently applied to protein digest followed by MS analysis. SILAC ratios are calculated by comparing intensities of heavy (red) to light (green) peaks of individual peptides (m, mass and z, charge). Direct ratios, reflecting relative changes, are determined thereof. The translatome is assessed by microarray analysis of RNA from polysome fractions. Polysomes are enriched by sucrose density gradient centrifugation of cells that have been treated with chloramphenicol. For transcriptome data, total RNA is isolated and applied to microarray analysis.

The “omics”-approach presented in this study compared relative changes in total mRNA and protein abundances. In addition, polysome fractions were collected to measure translational changes. The photo-oxidative stress response of the facultatively phototrophic bacterium *R. sphaeroides* was chosen as a model for bacterial stress responses. Exponentially growing cultures were treated with the artificial photosensitizer methylene blue in the presence of oxygen and high light intensities for the generation of singlet oxygen. The workflow of the three global approaches is illustrated in Figure 1 and will be explained in detail in the corresponding result sections. A comprehensive analysis allowed us to overlap all datasets (see Dataset S1) and this workflow will serve as proof-of-principle for the general suitability of the approach for bacterial stress responses.

### Dynamic features of biologically significant RNAs

It is regularly assumed that most of regulation is accomplished on transcript level. Up to now, no global transcriptome analysis of the photo-oxidative stress response in *R. sphaeroides* was conducted and there are only two studies referring to this topic indirectly [24], [26]. Here, total RNA, isolated before stress (reference) and at several time-points thereafter, was applied to microarray analysis to calculate relative changes in mRNA abundance (Figure 1, transcriptome). Data were collected for the short-term response (7 min) and for two later time-points (45 and 90 min) in biological duplicates. Reproducibility of experiments was high, as reflected by Pearson correlation *r* ranging between 0.80 and 0.98, and ratios showed a typical Gaussian distribution (Figure S1). A number of 65 mRNAs (1.5%) was significantly induced in expression after 7 min of stress (log_2_ ratio ≥0.8 and p-value <0.05; Table 1). At 45 min a higher portion of mRNAs was up-regulated (158 mRNAs (3.7%)), which was followed by a slight drop at 90 min (115 mRNAs (2.7%)). The photo-oxidative stress response obviously exhibited a peak of induction at time-point 45 min. Moreover, up-regulated mRNAs of the two late time-points showed a bigger overlap to each other than to mRNAs of the 7 min time-point (Figure S2), which was further confirmed by hierarchical clustering (data not shown). Therefore, mRNAs belonging to the short-term and/or the late stress response could be distinguished. However, the existence of a core set, comprising 51 mRNAs up-regulated at all time-points, was emerging (Figure S2). This applied to, e.g., the master regulators RpoE and RpoH2, photolyase PhrA, and the detoxifying glyoxalase II (GloB, RSP_0799). Numbers of down-regulated mRNAs (log_2_ ratio ≤−0.8) showed a similar trend as observed for up-regulated mRNAs, that is, most changes occurring at 45 min (Table 1).

**Table 1:** Number of quantified and regulated genes in individual approaches.

Experiment	Quantified mRNAs/proteins^1^	Up-regulated^2^		Down-regulated^2^	
		#	%	#	%
Total RNA 7 min	4289	65	1.5	23	0.5
Total RNA 45 min	4282	158	3.7	73	1.7
Total RNA 90 min	4282	115	2.7	57	1.3
Poly RNA 90 min	4258	129	3.0	24	0.6
SILAC 90 min	1214	68	5.6	45	3.7

1 The annotated *R. sphaeroides* 2.4.1 genome (http://img.jgi.doe.gov/cgi-bin/w/main.cgi) contains 4304 open reading frames. Numbers of mRNAs/proteins that were unambiguously quantified are depicted.

2 Numbers (#) and percentages (%) for regulated genes are given. Percentages were calculated relative to quantified mRNAs/proteins. Genes were considered to be regulated when log_2_ ratios (stress versus reference) were ≥0.8 (up-regulated) or ≤−0.8 (down-regulated) with p-values <0.05.

**Figure 2:**
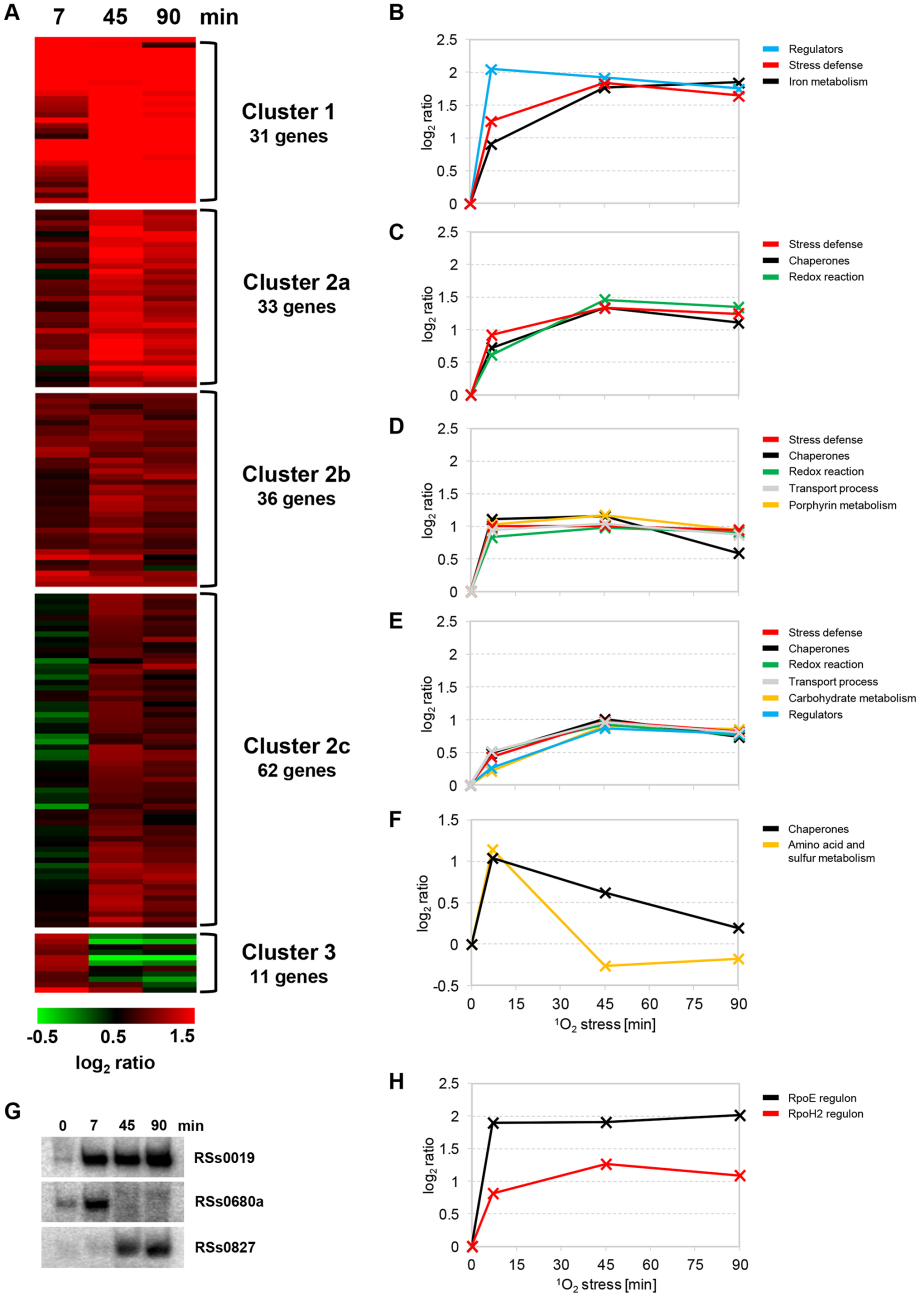
Expression kinetics of stress-related mRNAs and sRNAs. Relative changes of mRNAs upon singlet oxygen (^1^O_2_) stress were monitored by microarray analysis of total RNA at time-points 7, 45, and 90 min and depicted as log_2_ ratios. (A) All mRNAs with significant induction (log_2_ ratio ≥0.8 and p-value <0.05) at one of the three time-points were applied to clustering using MeV (Multi Experiment Viewer version 4.7.4) from the TM4 Microarray Software Suite [67], [68]. Clustering was based on k-means (KMC method) according to Euclidean distance with a maximum of 50 iterations. Changes were illustrated as heat-maps with a color code ranging from −0.5 (green) to 1.5 (red) log_2_ ratio. Columns represent time-points of the stress response and rows represent individual genes. (B–F) Up-regulated mRNAs shown in (A) were grouped together according to their function. Expression kinetics, representing the mean of log_2_ ratios, were calculated for functional groups of cluster 1 (B), cluster 2a (C), cluster 2b (D), cluster 2c (E), and cluster 3 (F). (G) Analysis of stress-induced sRNAs. Total RNA was isolated at the indicated time-points and applied to Northern blot analysis. RSs0019, RSs0680a, and RSs0827 were hybridized to radioactively labeled oligonucleotides and visualized by phosphoimaging. 5S rRNA was probed as a control for equal loading of samples (not shown). (H) Genes with described RpoE- and RpoH2-dependent promoters [28], [29], [72] were analyzed when they exhibited log_2_ ratios ≥0.8. The curves represent the mean of log_2_ ratios based on 12 mRNAs (RpoE, black line) and 42 mRNAs (RpoH2, red line). See supplementary Tables S1 and S2 for further information on regulated genes and their particular functions.

In a former study, expression kinetics of selected genes, measured by qRT-PCR, indicated that levels of individual mRNAs may peak at different time-points of the stress response [27]. In our study, we identified 173 mRNAs, showing significant induction at one of the three time-points, which could be grouped into three dynamic expression clusters according to k-means using the TM4 Microarray Software Suite (Figure 2A and Table S1). The biggest cluster was further divided into three sub-clusters (2a–2c). mRNAs within clusters 1, 2a, and 2b had an increased induction throughout the time-course, albeit induction was most pronounced at the two later time-points in several cases. This trend was even more obvious in cluster 2c. In contrast, the smaller cluster 3 comprises mRNAs which had a peak expression at 7 min. Functional groups were formed according to published data on the *R. sphaeroides* singlet oxygen stress response [27]–[31], combined with KEGG database searches (http://www.genome.jp/kegg/). Cluster 1 contains several genes with a function in stress defense or iron metabolism, like *phrA*, *gloB*, or bacterioferritin encoding *bfr* (Figure 2B). Interestingly, several genes for regulatory factors with a known or hypothesized role in the photo-oxidative stress response (namely *rpoE-chrR*, *rpoH2*, *rpoH1*, *ompR*) are found in cluster 1, which differs from cluster 2 in a stronger induction of corresponding mRNAs (several log_2_ ratios >1.5). Functional groups in cluster 2 relate to stress defense, chaperones, proteases, redox reaction, transport process, porphyrin and carbohydrate metabolism (Figures 2C–E). Several genes in cluster 2 are described for the first time to be part of the singlet oxygen response. They encode, e.g., the chaperones MoxR (RSP_1024), ClpA (RSP_2293), and GroES (RSP_2310), a thioredoxin (RSP_0725), and DNA ligase Lig2 (RSP_2413). In addition, genes for the transcriptional regulator Lrp (RSP_2719) and a TetR family regulator (RSP_2853) were newly identified as stress-responsive (see Table S5). In contrast to cluster 1 and 2, genes of cluster 3 clearly belong to the short-term response and their products mainly have a function in amino acid and sulfur metabolism, like phosphoglycerate dehydrogenase SerA (RSP_1352) and sulfite reductase CysI (RSP_1942). In addition, several chaperones can be found in cluster 3 (Figure 2F).

Many genes of the photo-oxidative stress response are transcriptionally regulated by the two alternative sigma factors RpoE and RpoH2. Upon stress, RpoE is ultimately activated by release from its anti-sigma factor ChrR, while RpoH2 is produced downstream of RpoE [27], [29], [32]–[34]. We therefore assumed that members of the two sigma factor regulons exhibit different kinetics. Indeed, genes within the RpoE regulon were strongly up-regulated already after 7 min of stress, while RpoH2-dependent genes showed a delayed induction and full expression was not observed before 45 min (Figure 2H). Assignment of the RpoH2 regulon was based on genome-wide predictions for conserved promoter sequences [28], [29]. Very recently, an alternative RpoH2 regulon was defined according to expression profiles and ChIP-chip experiments [35], which we used to compile expression kinetics from our data sets. Intriguingly, results for both RpoH2 regulons were nearly congruent with each other (Figure S2).

We were also interested in expression patterns of sRNAs, which are important post-transcriptional regulators in bacteria. Microarrays designed for this study included probes for 144 verified and potential sRNAs that have been identified in our group [36], [37]. sRNA expression kinetics resembled those described above for mRNAs and similar dynamic clusters could have been formed (Figure S2). Northern blot validation clearly demonstrated, that the RpoE-dependent RSs0019 sRNA is highly expressed during the whole time-course of the experiment, while expression of the RpoH2-dependent RSs0680a sRNA exhibited a peak at 7 min (Figure 2G). Investigation of late time-points revealed that RSs0827 is strongly induced only after prolonged singlet oxygen stress (Figure 2G). RSs0827 was recently shown to respond to iron limitation [38] and can now be placed on the growing list of (photo-) oxidative stress inducible sRNAs in bacteria.

### SILAC-based proteomics reveal changes in protein abundance

The SILAC method was invented to enable quantitative proteomics of complex protein samples by MS [13]. For the photo-oxidative stress response of *R. sphaeroides*, we made use of an indirect quantification approach by applying a heavy standard generated by SILAC-labeling. *R. sphaeroides* cultures were supplemented with the heavy amino acid ^13^C_6_-lysine (Lys6), allowed to grow, and subsequently diluted several times into fresh Lys6-containing medium to achieve complete labeling of proteins (incorporation rate of 96%, Figure S3 and Dataset S2). The heavy standard represents a protein mixture obtained from fully labeled cultures grown under semi-aerobic, aerobic, and singlet oxygen stress conditions to cover a broad protein pattern from various physiological states. Therefore the heavy standard was referred to as “bacterial SILAC standard” according to the super-SILAC mix of human breast cancer cells [39]. The bacterial SILAC standard was mixed in a 1∶1 ratio with protein samples from unstressed (reference) and stressed cultures, which were grown in presence of the light amino acid ^12^C_6_-lysine (Lys0). Based on intensity differences between heavy and light peptide peaks derived from LC-MS/MS analysis, SILAC protein ratios were calculated. To verify SILAC protein quantification, two protein digestions (insol and ingel digestion) were performed in biological duplicates (n = 4). Pearson correlation *r* between replicates ranged between 0.86 and 0.94, which reflects the high reproducibility of the SILAC approach (Figure S4). Next, mean SILAC ratios of the quadruplicates were divided to calculate direct protein ratios between stressed and unstressed cultures ((heavy standard/reference)/(heavy standard/90 min ^1^O_2_)), which were statistically verified by determining p-values (Figure 1, proteome).

**Figure 3:**
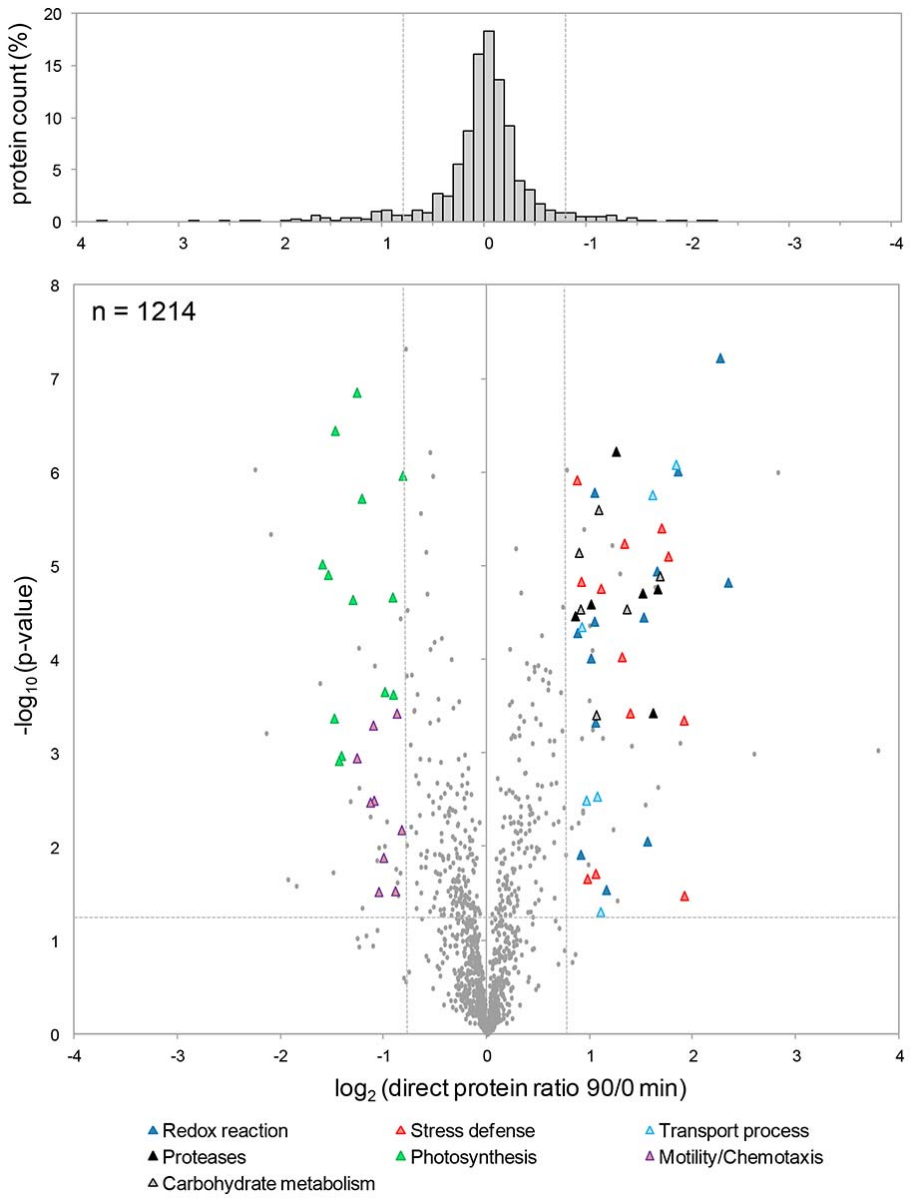
Accurate quantification of protein changes by SILAC. Relative changes in protein abundance after 90 min of singlet oxygen stress were determined by an indirect quantification approach using a heavy standard labeled with ^13^C_6_-lysine (Lys6). Protein mixtures were digested and used for MS analysis (see Materials and Methods). In the central volcano plot the direct ratios (log_2_) of 1214 quantified proteins of quadruplicates were plotted against negative logarithmized p-values (log_10_). The histogram on the top shows log_2_ protein ratio distributions (Gaussian distribution). Up- and down-regulated proteins were grouped according to their functions which relate to stress defense (red triangles), proteases (black triangles), redox reactions (dark blue triangles), carbohydrate metabolism (grey triangles), transport processes (light blue triangles), photosynthesis (green triangles), and motility/chemotaxis (purple triangles). See supplementary Table S3 for further information on regulated proteins and their particular functions.

The SILAC approach identified 1538 proteins with at least two peptides, from which 1214 proteins were quantified (Figure 3 and Dataset S2). The distribution of the log_2_ protein ratios exhibited a Gaussian-like curve, which underlines the reliability of the approach. At time-point 90 min of the photo-oxidative stress response, 68 proteins (5.6%) were significantly up-regulated (log_2_ ratio ≥0.8, p<0.05; Table 1 and S3), while 45 proteins (3.7%) were significantly down-regulated (log_2_ ratio ≤−0.8, p<0.05; Table 1 and S3). Several of the up-regulated proteins have a function in stress defense, redox reactions, carbohydrate metabolism, and transport processes or are acting as proteases (Figure 3), which corresponds to observations made for mRNAs changed during the photo-oxidative stress response (Figure 2). Among the down-regulated proteins, two major groups relate to photosynthesis as well as motility/chemotaxis (Figure 3). Altogether, these data demonstrate that the SILAC approach highlights proteins with altered abundances upon stress with high confidence.

### The SILAC approach identifies new open reading frames

**Figure 4:**
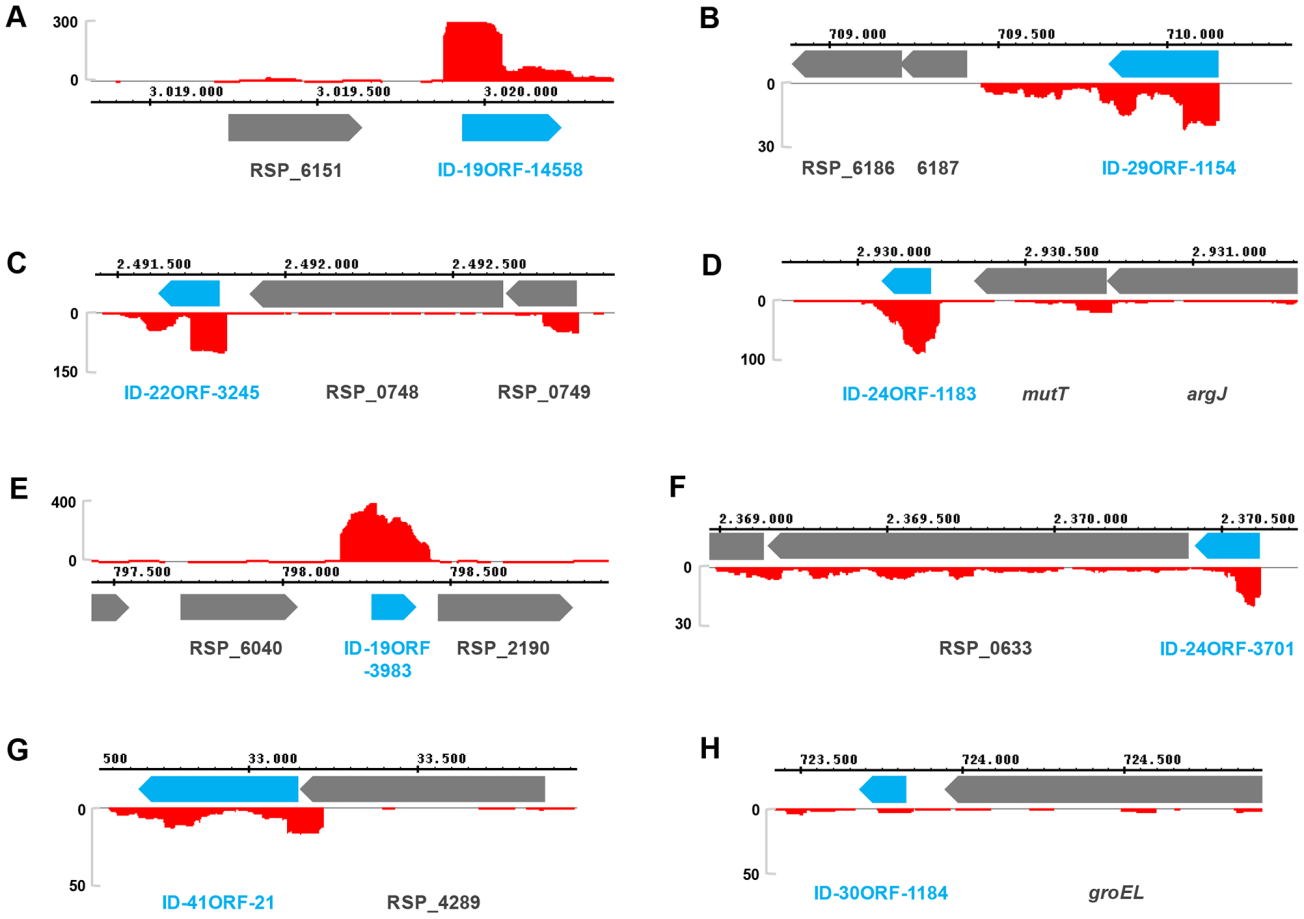
RNA-seq validation for ORFs newly identified by SILAC. ORFs detected by SILAC-based MS were compared to deep sequencing data (RNA-seq). (A–H) Integrated Genome Browser (Affymetrix) screenshots depict gene loci with nucleotide positions referring to the plus-strand. RNA-seq data are visualized as numbers of cDNA reads per nucleotide (red plots). Grey and blue arrows represent annotated and new ORFs, respectively. For further details on new ORFs see Table S4.

In contrast to microarrays, which are designed according to the available information of the annotated genome, the SILAC approach presented here is annotation-independent. Consequently, several peptides were identified which potentially represent new open reading frames (ORFs) (Table S4). In order to validate these putative ORFs on RNA level, we inspected RNA-sequencing (RNA-seq) data available in our group (these data are based on deep-sequencing of RNA from exponentially growing wild-type cultures under semi-aerobic conditions and will be published elsewhere). First, RNA-seq data were screened for the presence of cDNA reads at the particular position of a new ORF. When cDNA reads gave reliable coverage for the gene locus, apparent transcriptional start sites were related to potential translational starts. In 13 out of 19 cases, RNA-seq strongly supports the SILAC data, while for the remaining examples sequencing coverage was not sufficient (Table S4). For some new ORFs it is very likely that they represent stand-alone genes (Figure 4A–D), while others are located between or in front of genes and might therefore be part of operons (Figure 4E–F). BLAST searches suggested functions in, e.g., transcription regulation (ID-19ORF-14558 and ID-29ORF-1154) or translation (ID-24ORF-1183). Intriguingly, two proteins derived from potentially new ORFs were up-regulated under singlet oxygen stress. This applies to gene ID-41ORF-21 (log_2_ protein ratio 0.85), which shows homology to uridylate kinases and might therefore have a role in cell division [40]. RNA-seq data suggested ID-41ORF-21 to be transcribed from its own promoter internal to RSP_4289 (Figure 4G). The new ORF ID-30ORF-1184 (log_2_ protein ratio 0.78) is located downstream of a *groEL* gene and might be functionally related to chaperone functions. Unfortunately, cDNA read coverage is too low to assume co-regulation of ID-30ORF-1184 with *groEL* (Figure 4H).

As a conclusion, our SILAC approach is a powerful tool for both the identification of unknown ORFs and simultaneous quantification of corresponding protein levels. Its application to any stress response in bacteria will give valuable and new insights.

### Are changes in the transcriptome transferred to the proteome level?

**Figure 5:**
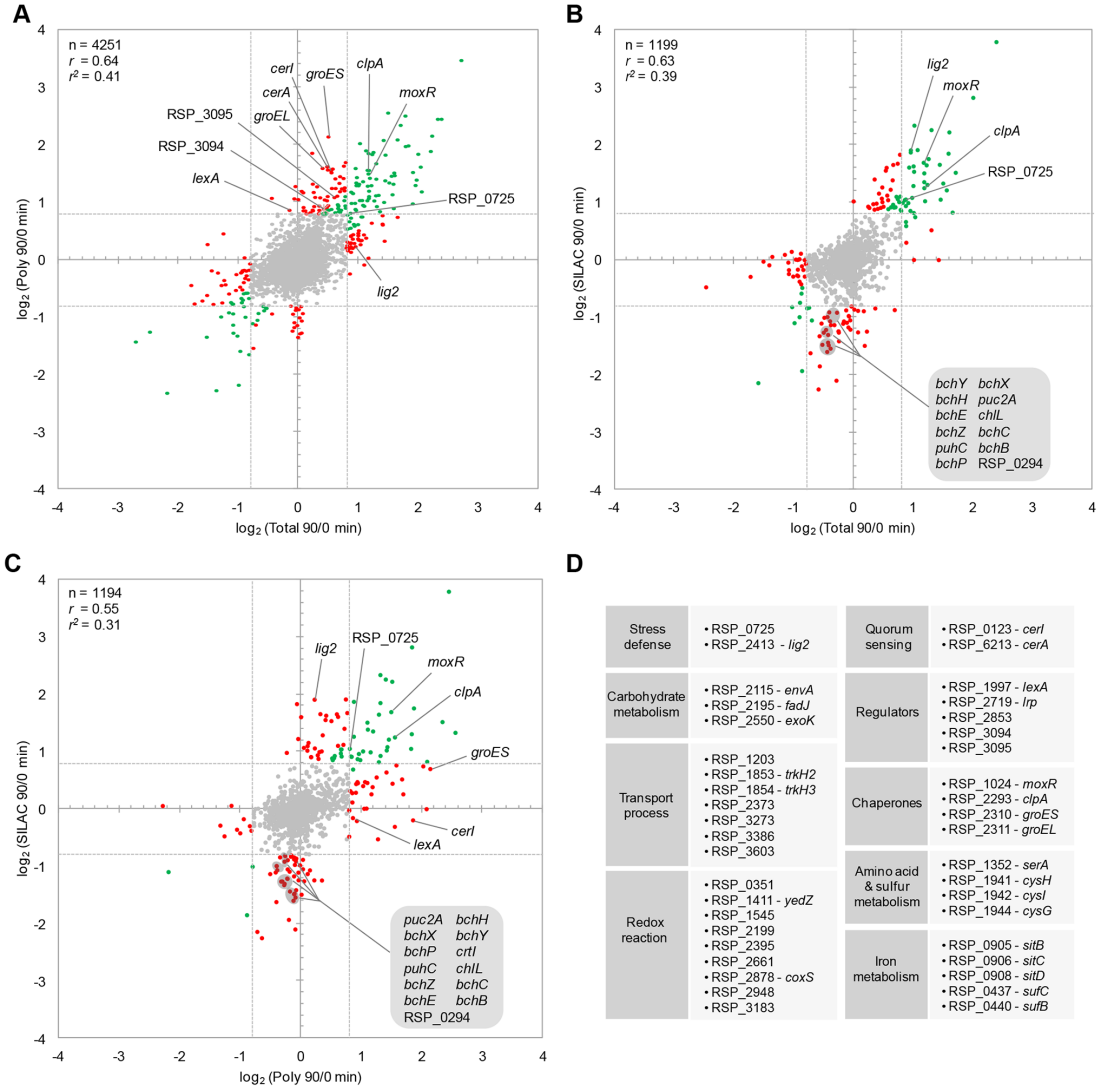
Correlation between global approaches. Scatter-plots represent pairwise comparisons of log_2_ ratios between (A) transcriptome (Total 90/0 min) and translatome (Poly 90/0 min), (B) proteome (SILAC 90/0 min) and transcriptome, (C) proteome and translatome. Number (n), Pearson correlation (*r*), and squared correlation (*r^2^*) of shared features are given for every comparison. It is indicated for up-regulated (log_2_ ratio ≥0.8) and down-regulated (log_2_ ratio ≤−0.8) features whether changes are unidirectional (log_2_ ratio difference between approaches <0.4, green spots) or biased (log_2_ ratio difference ≥0.4, red spots). (D) Functional grouping of genes that were newly identified to be stress-responsive by the integrative approach. For a complete list of genes and information on their function see Table S5.

One of the main issues to be addressed in this study is the question of how and to which extent changes at transcriptome level impact protein abundances. In this context, the translatome was of major interest since it represents the mRNA-protein interface. The translatome was assessed by microarray analysis of mRNAs in polysome fractions with high reproducibility between biological duplicates (Pearson correlation *r* = 0.91, Figure S1). Polysomes were enriched by sucrose density gradient centrifugation of crude extracts after chloramphenicol treatment of cells (Figure 1 and S5). As for total RNA and proteins, changes of polysomal mRNAs after 90 min of stress were calculated relative to the reference (no stress). Transcriptomic, translatomic, and proteomic data sets at time-point 90 min were correlated to each other and visualized as scatter-plots (Figure 5). The transcriptome and translatome showed a fairly high correlation (*r* = 0.64; n = 4251) with a major distribution of data-points in the middle of the plot, representing genes that exhibited no or only minor changes. However, 98 genes were up-regulated on both transcriptional and translational level. Besides well-known genes, several candidates were newly identified for the photo-oxidative stress response, which is exemplified by *moxR* and *clpA*, encoding chaperones, and thioredoxin RSP_0725 (Figure 5A). In contrast, 51 genes were translationally triggered without showing a comparable increase on transcriptome level (log_2_ ratio difference of at least 0.4; Figure 5A). In this group several genes could be linked to the photo-oxidative stress response for the first time, as shown for regulators (*lexA* and the sigma factor/anti-sigma factor operon RSP_3095-94), chaperones (*groES* and *groEL*), as well as quorum sensing (*cerI* and *cerA*).

A different picture emerged when correlating the proteome data; overall correlation to the transcriptome was fairly high (*r* = 0.63; n = 1199), as indicated by genes that were up-regulated in both approaches. However, a group of 43 genes was decreased in protein abundance (log_2_ ratio ≤−0.8), while not changed on transcriptome level to the same extent. A major portion of this group has a function in photosynthesis (12 genes; Figure 5B). A similar observation was made when comparing the proteome and translatome (*r* = 0.55; n = 1194); 13 photosynthesis genes with decreased protein levels were not depleted in polysomes. Interestingly, 31 genes showed an increased emergence in the polysome fraction after stress without changing protein abundance, as observed for the regulator LexA, the autoinducer synthesis protein CerI, and the chaperone GroES (Figure 5C).

In general, it emerged that ∼41% of mRNA variance within polysomes (translational effects) can be explained by changes in the transcriptome (*r^2^* = 0.41, Figure 5A). However, only ∼31% of variance in protein levels could be assigned to changes in translation (*r^2^* = 0.31, Figure 5C), although ∼39% of this variance could be explained by mRNA levels (*r^2^* = 0.39, Figure 5B). It appeared that, beside unidirectional effects, substantial regulation occurred separately on all levels. This can be exemplified by expression changes of *lig2*, encoding a newly identified stress-related DNA ligase. Protein and mRNA levels are both induced, although polysome association is rarely changed. A possible explanation would point to parallel regulation on both transcriptional and post-translational level without influencing translation itself. Since technical limitations cannot be excluded, these examples need further validation and present interesting subjects for future studies.

### Genes within operons exhibit polar expression patterns

**Figure 6:**
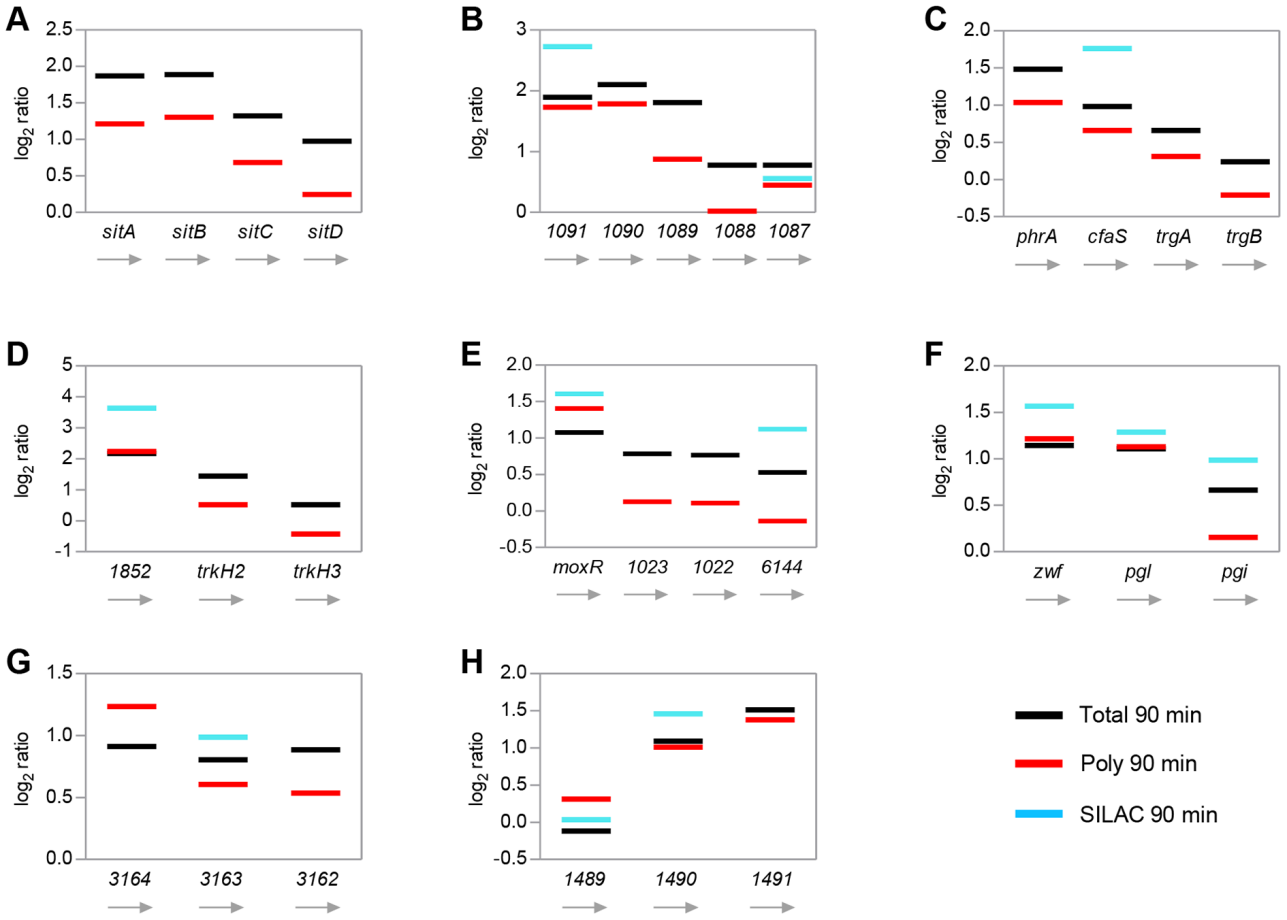
Polarity of stress-induced operons. Graphs representing relative changes for selected operons after 90 min of singlet oxygen stress. Log_2_ ratios were derived from transcriptomic (Total 90 min, black bars), translatomic (Poly 90 min, red bars), and proteomic (SILAC 90 min, blue bars) data sets. Orientation of genes within operons is depicted from the left to the right, irrespective of their location in the genome, with the leftmost gene representing the first gene in the operon. Gene numbers refer to corresponding RSP-numbers. Operons were selected according to following categories: transport process and iron metabolism (A), RpoE-dependent (B, C, D), stress defense (C), transport process (D), chaperone (E), RpoH2-dependent (F, G), carbohydrate metabolism (F), redox reaction (G), protease (H).

In the genome-reduced bacterium *Mycoplasma pneumoniae* almost half of the polycistronic operons show a staircase-like expression [41]. Furthermore, operons can be divided into suboperons with dynamics depending on environmental conditions. It was assumed that this phenomenon is widespread in bacteria, which motivated us to inspect expression patterns of stress-induced operons of *R. sphaeroides* carefully. Notably, relative changes between time-points rather than relative expression levels at one time-point were matched. When comparing changes of total RNA levels after 90 min of singlet oxygen stress (transcriptome), a staircase-like pattern emerged as a common feature. In most cases, log_2_ ratios decreased from the first to the last gene in an operon (Figure 6A–F), from now on referred to as 5′ polarity. Other operons exhibited no polarity (Figure 6G) or featured a 3′ polarity (Figure 6H). At translatome level orientation of polarity and even particular ratios were quite similar to transcriptomic data, indicating that transcriptional polarity impacts translation. There was one exception: genes within the RSP_3164-62 operon were equally regulated at transcriptome level, but exhibited a 5′ polarity at translatome level (Figure 6G). Furthermore, in some cases it was indicated that both 5′ and 3′ polarity are transmitted to protein levels (Figure 6B, E, F, H), for the remaining operons proteome data were not complete enough to give a reliable picture. When comparing expression levels at time-points 0 min and 90 min separately, it emerged that polarity is more pronounced after stress (Figure S6), which indicates that operon polarity might be inducible.

## Discussion

The simple concept of transcriptional regulation being the key determinant of gene expression fails to explain the observed flexibility of bacterial adaptation. Steadily increasing numbers of investigations reveal that complexity of bacterial regulation nearly resembles that of eukaryotic cells [42]. To fully understand how bacteria explore complex gene expression control in response to stress, changes have to be measured on the system level, and in order to appreciate post-transcriptional and post-translational events, system biology studies need to consider multiple layers of regulation. The “omics” approach presented here compared relative changes in abundance of total mRNAs, mRNAs captured in polysomes for translation, and proteins after sub-lethal stress. The singlet oxygen stress response of *R. sphaeroides* served as an example to validate our approach. While mRNA levels can be easily quantified by microarray analysis on an almost-global scale, comprehensive proteomics relies on state-of-the-art MS combined with accurate quantification methods. Here we used an indirect quantification approach by applying a heavy standard generated by SILAC. The bacterial SILAC standard represents several physiological states and is therefore similar to the super-SILAC approach applied to mammalian systems [12], [39]. We successfully identified 1538 proteins, from which 1214 could be accurately quantified with high reproducibility (Figure 3). In *Bacillus subtilis*, which has a genome size comparable to *R. sphaeroides*, 1928 proteins were identified by MS during logarithmic growth, representing more than 75% of genes expected to be expressed [19]. In *R. sphaeroides* the number of expressed genes under aerobic and singlet oxygen stress conditions is not known, and therefore, estimations of protein coverage are difficult. However, our approach seems to be as comprehensive as comparable studies. Several new open reading frames were identified, which were partly validated by RNA-seq (Figure 4). Two of the newly identified genes showed stress-dependent expression and thus extend the list of singlet oxygen responsive genes. During the last years several studies applied differential RNA-seq to identify sRNAs and to globally map transcriptional start sites in bacteria [43], [44]. In the near future, MS-based protein identification/quantification together with RNA-seq in a combinatory approach may be the experimental tool of choice to re-annotate genomes and to generate expression maps in various organisms.

Bacterial stress responses are highly dynamic in many ways. Up to date no studies comparing the transcriptome of *R. sphaeroides* in presence of singlet oxygen to non-stress conditions were presented and kinetics for mRNA levels or protein synthesis rates after onset of singlet oxygen stress were available only for few examples [25]. With the data provided here, stress-related genes could be globally identified and grouped according to their expression patterns, which also allowed us to distinguish short-term responses from late adaptation processes. For example, some genes involved in sulfur metabolism exhibit a pulse expression during the first minutes of the singlet oxygen stress response in *R. sphaeroides* (Figure 2F). This pulse expression might be explained by an incoherent feed-forward loop [45], which either consists of protein regulators or also includes sRNAs, as recently described for regulation of photosynthesis genes in *R. sphaeroides* [46]. Vice versa, regulators may not only cause pulse-expression but are subject to pulse-expression themselves, as monitored for the RSs0680a sRNA (Figure 2G). Quantification of transcripts from alternative sigma factor regulons revealed that individual activation characteristics of the sigma factors under stress will ultimately determine expression dynamics of downstream genes (Figure 2H). While RpoE-dependent genes are constantly induced, RpoH2-dependent genes show a delayed induction and are most likely important for late adaptation processes.

Furthermore, our study revealed transcripts/proteins with singlet oxygen-dependent abundance, which were not recognized as stress-dependent before (Figure 5D and Table S5). Interestingly, the new members of singlet oxygen-dependent genes comprise several transcriptional regulators including a sigma factor/anti-sigma factor system and the *cerI* and *cerA* genes [47] suggesting that singlet oxygen affects quorum sensing. Our study also extends the list of genes for chaperons, iron metabolism, and sulfur metabolism which respond to singlet oxygen stress.

Another dynamic expression feature in bacteria applies to operon polarity, which was described for *Mycoplasma pneumoniae* [41]. The observation of a staircase-like expression pattern, with the first gene in an operon showing the highest expression, might be explained by transcriptional-translational coupling and cross-talk between the responsible machineries in such a way, that transcription is interrupted whenever ribosomes hit a stop codon [42]. Our results reveal that the particular position of a gene within a stress-inducible operon not only impacts expression but also the degree of induction, with the first gene mainly be induced to the highest degree, what we refer to as 5′ polarity (Figure 6). Several of the stress-inducible operons in *R. sphaeroides* exhibit a clear 5′ polarity on both transcriptome and translatome level. Interestingly, polarity of transcript levels is clearly more pronounced after stress indicating that polar expression is stress-induced (Figure S6). Unidirectional behavior of transcriptional and translational effects was regularly observed for large sets of genes, as e.g. under severe stress in yeast or in halophilic archaea [7], [10]. However, 5′ polarity may only be achieved by post-transcriptional mechanisms, including sRNAs or RNA secondary structures, leading to non-correlated induction (Figure 6G). It was assumed that expression polarity is compensated for on protein level [6]. For the stress-inducible operons presented here it appears that polarity on transcriptome/translatome level also entails polar changes of protein abundance, which argues against compensation in these particular examples. Finally, the occurrence of 3′ polarity (Figure 6H) implies that several distinct mechanisms underlie the polarity phenomenon. Differential stability of polycistronic mRNAs and other post-transcriptional regulation events might explain the observed diversity. However, bacteria obviously exploit the order of genes within operons to fine-tune gene expression.

We systematically investigated the transcriptome, translatome, and proteome to explore global correlations and estimate the importance of particular modes of regulation upon stress in bacteria. Direct dependence between transcript levels and translation is observed for ∼41% of all mRNAs (*r^2^* = 0.41, Figure 5A), while ∼59% may be subject to post-transcriptional events which alter translation irrespective of mRNA levels. A predominant class of regulators that act on the post-transcriptional level are *trans* encoded sRNAs [48]. Regulons controlled by sRNAs may be as large as described for GcvB that impacts on ∼1% of all transcripts through a conserved binding domain [49]. In addition to sRNAs, mRNA stability, mRNA structure, and RNA-binding proteins play important roles for translation. Here we show that translational control is a fundamental way to globally induce genes upon stress in bacteria, which is similar to observations in yeast [10]. In *R. sphaeroides* this is reflected by the fact that after 90 min of singlet oxygen stress 3.0% of genes are up-regulated on translatome level compared to only 2.7% on transcriptome level (Table 1). Assessing the translatome should therefore be regularly considered when investigating cellular stress responses, either in addition to or as an alternative to transcriptome studies. The SILAC approach delivers further valuable insights into global regulatory events. The observation that only ∼31% of protein changes can be matched to translational changes (*r^2^* = 0.31, Figure 5C) is unexpected, but can be explained by altered turn-over and degradation of proteins upon stress. Proteins are a major target for singlet oxygen in cells and damaged or fragmented proteins need to be removed [50]–[52]. The stress-dependent induction of several proteases is consistent with increased protein turn-over in presence of singlet oxygen but their particular roles in stress-dependent protein turn-over need experimental verification. The decline of photosynthetic proteins points to post-translational regulation by degradation, which may be achieved by the above-mentioned stress-induced proteases. Since reactions of singlet oxygen with amino acids can lead to depletion of the amino acid pool [53], selective degradation of photosynthetic proteins would both replenish the amino acid pool and avoid additional singlet oxygen generation in photosynthetic complexes. However, various post-translational events might cause that the ribosome coverage is only poorly correlated to protein abundance and we assume that comparing translation to protein synthesis, e.g. by using pulsed SILAC [54], would be a valuable experiment to achieve higher correlations.

Despite all the challenging open questions, our integrative approach delivered a comprehensive list of genes that are relevant to the singlet oxygen response and provided conclusive evidence for their regulation. For example, for quorum sensing genes (*cerI*, *cerA*) as well as for several genes encoding regulators (*lexA*, RSP_3095-94) substantial regulation seems to occur only on the translational level. This might be the reason why these genes have been overseen in former studies. The current data set will encourage detailed studies on newly identified players of the bacterial response to singlet oxygen.

## Materials and Methods

### Growth conditions and stress experiments

For all experiments conducted in this study *Rhodobacter sphaeroides* wild-type 2.4.1 [55] was cultivated at 32°C in minimal salt medium with malate as carbon source [56]. Pre-cultures were grown in Erlenmeyer flasks with continuous shaking at 140 rpm, resulting in a dissolved oxygen concentration of approximately 25 mM (semi-aerobic conditions). The amino acid ^12^C_6_-lysine (Lys0; Sigma-Aldrich) or its stable isotope counterpart ^13^C_6_-lysine (Lys6; Silantes) were added to the cultures in a final concentration of 50 µg ml^−1^. Pre-cultures were grown until an optical density at 660 nm (OD_660_) of ∼0.8–1.0 was reached and subsequently diluted into fresh Lys0/Lys6-containing medium in a concentration of 0.5% (v/v), which was repeated two times. Cultivation of *R. sphaeroides* in the presence of Lys6 over several generations enabled full labeling of proteins with stable isotopes (Figure S3). Lys0-treated cultures were used for regular stress experiments, while Lys6-labeled cultures were applied to generate a heavy standard for SILAC-based mass spectrometry. For stress experiments, semi-aerobic pre-cultures were diluted with Lys0/Lys6-containing medium to an OD_660_ of 0.2 and methylene blue was added in a final concentration of 0.2 µM. Cultures were gassed with air in flat glass bottles, resulting in a dissolved oxygen concentration of approximately 180 mM (aerobic conditions), and grown in the dark to an OD_660_ of 0.4. Singlet oxygen was generated by applying high light (800 W m^−2^) with a white light halogen bulb as described [23]. Samples, collected at the indicated time-points, were rapidly cooled on ice and centrifuged at 10,000*g* for 10 min at 4°C. For polysome preparation, cells were treated with chloramphenicol in a final concentration of 0.1 mg ml^−1^ for 5 min at 32°C before harvesting.

### SILAC-based mass spectrometry

Heavy labeled and non-labeled cells were completely lysed in SDS-Buffer (4% SDS in 100 mM Tris/HCl pH 7.6) and shortly heated at 95°C. Sonication was performed for DNA sharing prior to sample centrifugation at 16,000*g* for 5 min. Protein concentration of the clear supernatant was measured by DC protein assay (Biorad) to mix labeled and non-labeled protein samples in the same amount. To reduce sample complexity for MS-analysis, proteins were separated by SDS-PAGE (NuPAGE 4%–12% Bis-Tris gel, Invitrogen) and stained with Colloidal Blue Staining Kit (Invitrogen). Each lane was cut into 14 gel pieces for in-gel digestion as described [57]. In brief, proteins were reduced by 50 mM dithiothreitol (DTT), alkylated with 550 mM iodoacetamide and digested with the endopeptidase Lys-C (enzyme to protein ratio 1∶100, Wako). After digestion and elution, peptides were desalted by stop and go extraction (STAGE) tips [58]. Next to in-gel digestion, samples were also digested in solution as described [59].

For LC-MS/MS, a nano liquid chromatography (LC) system (Thermo Fisher Scientific) was coupled to a LTQ-Orbitrap Velos or a Q-Exactive mass spectrometer (Thermo Fisher Scientific) via a nanoelectrospray source (Proxeon). Fused silica emitter were packed in-house with C18-AQ RepoSil-Pur (3 µm, Dr. Maisch GmbH) and used as columns for reverse-phase chromatography to separate peptides by a linear gradient of 5–30% acetonitril with 0.5% acetic acid for 150 or 240 min at a flow rate of 200 nl min^−1^. After elution, peptides were ionized and transferred to gas-phase by electrospray ionization (ESI) to enter the mass spectrometer. For measurements with the LTQ-Orbitrap Velos mass spectrometer, full MS scan spectra (m/z = 300–1650) were acquired in the Orbitrap with a resolution of R = 60,000 after accumulation of 1,000,000 ions. The 15 most intense peaks from full MS scan were isolated and fragmented in the linear ion trap after accumulation of 5,000 ions. Fragmentation of precursor ions was performed using CID (35% normalized collision energy) prior to acquisition of MS/MS scan spectra. Q-Exactive measurements were performed as described [60]. The 10 most intense peaks were selected and fragmented by higher energy collisional dissociation.

Analysis of raw data was performed by the MaxQuant software package (version 1.2.2.9) as described [61]. Database searches were performed with the Andromeda search engine against a house-made *R. sphaeroides* 2.4.1 database. The two chromosomes and five plasmids of *R. sphaeroides* 2.4.1 were translated into protein sequences using EMBOSS Transeq [62]. Translation was performed for all six reading frames. A unique identifier indicating the sequence position and frame was generated and assigned to each resulting open reading frame (ORF) longer than six amino acids. The generated ORF database was combined with all public available protein sequences for *R. sphaeroides* 2.4.1 and then used for the peptide identification step. By applying a decoy approach we determined the false discovery rate (FDR) to be smaller than 1%. After peptide identification, database entries belonging either to the *de novo* generated ORF set or to the public available annotated proteins were clustered into protein groups (MaxQuant). Groups lacking an annotated member were assumed to be potentially new coding sequences and were selected for further investigation.

Detection and quantification of SILAC pairs was performed by MaxQuant using following parameters: Lys-C as digesting enzyme with a maximum of two missed cleavages, carbamidomethylation of cysteins as fixed modification, oxidation of methionine and acetylation of the protein N-terminus as variable modifications, SILAC amino acid labeling: Lys6. Maximum mass deviation was set to 7 ppm for the peptide mass and 0.5 Da for MS/MS ions. For identification of peptides and proteins a FDR of 1% were used and only peptides with minimum of six amino acids length were considered for identification. For SILAC analysis, two ratio counts were set as a minimum for quantification. Bioinformatic analysis was performed with Perseus (version 1.3.0.4) to calculate p-values with a Benjamini-Hochberg multiple testing correction based on a FDR threshold of 0.05.

### Preparation of polysomes

Polysomes were prepared basically as described elsewhere [63]. Cell pellets derived from 200 ml chloramphenicol-treated cultures were resuspended in 4 ml cold polysome buffer (P buffer: 10 mM Tris pH 7.6, 60 mM NH_4_Cl, 3 mM Mg(CH_3_COO)_2_) and used for lysis by gentle sonication in an ice bath. The cell debris was removed by centrifugation at 15,000*g* for 10 min at 4°C. Three ml supernatant were applied to sucrose density gradients, which were prepared by layering 3 ml 0.9 M sucrose on 3 ml 1.8 M sucrose (as solutions in P buffer) in 13.2 ml polyallomer thinwall tubes (Herolab). Ultracentrifugation (200,000*g*, 16 hours, 4°C) was carried out using a SW41-Ti rotor (Beckman Coulter) in a Discovery 90 ultracentrifuge (Sorvall). The gradient was divided into nine fractions and used for downstream validation (Figure S5). The pellet representing the polysome fraction was layered with 100 µl P buffer and incubated on ice for up to 3 hours to enable complete resuspension of polysomes. Polysome fractions were used for RNA isolation.

### RNA isolation and quality assignment

RNA from both crude extracts and polysome fractions was isolated using the hot phenol method [64], followed by one (Northern) or two (microarray) chloroform-isoamylalcohol treatments and precipitation with sodium acetate and ethanol. RNA was resolved in RNase-free water (Roth) and concentrations were determined at a NanoDrop 1000 Spectrophotometer (Peqlab). RNA for Northern blot detection was directly used after isolation, while RNA for microarray analysis was further processed. Total RNA for microarrays was treated with DNaseI (Invitrogen) to remove contaminating DNA, followed by purification using the RNeasy MinElute Cleanup Kit (Qiagen). RNA from polysome fractions was purified accordingly. Absence of DNA was monitored by PCR using Taq DNA Polymerase (Qiagen) and primers RSP0799-A (5′-GAA CAA TTA CGC CTT CTC) and RSP0799-B (5′-CAT CAG CTG GTA GCT CTC) [23]. Polyacrylamide-gels (10%, v/v) containing 7 M urea were prepared to assess RNA quality.

### Microarray analysis

For gene expression studies, isolated RNA was hybridized to Custom Gene Expression Microarrays from Agilent Technologies (8x15K; ID: 027061) designed for *R. sphaeroides* wild-type 2.4.1 [65]. The arrays contain oligodeoxynucleotide probes (60-mers) for 4304 open reading frames, according to genome annotations available on the IMG server (Integrated Microbial Genomes; img.jgi.doe.gov/cgi-bin/w/main.cgi), and for 144 putative sRNAs identified in our group [36], [37]. Two µg RNA from reference (no stress) and stress samples were chemically labeled with Cy5 and Cy3, respectively, using the ULS Fluorescent Labeling Kit for Agilent arrays (Kreatech) and competitively hybridized to arrays (two-color microarrays). Fragmentation of labeled RNA, hybridization to arrays, and washing was performed using the Gene Expression Hybridization and Wash Buffer Kits according to the specifications of Agilent. Hybridization was performed at 65°C for 17 hours. Read-out files for arrays were generated with the Agilent DNA microarray scanner, followed by compilation of raw median fluorescence values using the Feature Extraction Software (Agilent). Within-array normalization according to LOESS was accomplished with the Bioconductor package Limma for R [66]. Those values were retained that exhibited an average signal intensity (A-value: 1/2 log_2_ (Cy3×Cy5)) above background, as specified by Agilent control probes present on each array (Poly 90 min A≥10.44; Total 7 min A≥10.27; Total 45 min A≥10.61, Total 90 min A≥10.45). Fold changes were calculated from remaining values as log_2_ ratios (Cy3/Cy5). Data shown in this study represent the results from two individual microarrays (biological replicates), each containing a pool of three independent experiments for each sample. Statistical analysis was performed by Perl Statistics modules. Targets having p-values <0.05 and log_2_ ratios ≥0.8 or ≤−0.8 were assumed to represent deregulated candidates. For expression cluster analysis, log_2_ ratios were imported to MeV (Multi Experiment Viewer version 4.7.4) from the TM4 Microarray Software Suite [67], [68] and visualized as heat-maps. Clustering was based on k-means (KMC method) according to Euclidean distance with a maximum of 50 iterations.

The data discussed in this publication have been deposited in NCBI's Gene Expression Omnibus [69] and are accessible through GEO Series accession number GSE42244 (http://www.ncbi.nlm.nih.gov/geo/query/acc.cgi?acc=GSE42244).

### Northern blot detection

For detection of sRNAs, 10 µg of total RNA were separated on 10% (v/v) polyacrylamide-gels containing 7 M urea and 1× TBE. Gel runs were performed at 300 V for approximately 3 hours in 1× TBE. RNA was transferred to SensiBlot Plus Nylon Membranes (Fermentas) by semi-dry electroblotting in 1× TBE (250 mA, 3 hours), followed by cross-linking with UV light. 5′ end-labeling of oligodeoxynucleotides with [γ-^32^P]-ATP as well as hybridization, washing, and documentation of membranes was performed as described elsewhere [36]. Oligodeoxynucleotides for probe generation were: p-0019 (5′-GAG ATA GCT CAT CGG TCA GGT CC), p-0680a (5′-CGT CGC CGC TGC TGC TAC AGG TC) [36], and p-0827 (5′-GGA CAG TGA AGG TAG AAC GG) [38].

### RNA-sequencing

RNA for sequencing was isolated as described for microarray analysis. *R. sphaeroides* 2.4.1 cultures were grown under semi-aerobic conditions to a final OD_660_ of 0.4. The cDNA libraries were prepared at Vertis Biotechnology AG (Germany). For this, the RNA samples were poly(A)-tailed by poly(A) polymerase. After that, the 5′-PPP residues were removed using tobacco acid pyrophosphatase (TAP) followed by the ligation of the RNA adapter to the 5′-phosphate of the RNA. First-strand cDNA synthesis was performed using an oligo(dT)-adapter primer and the M-MLV reverse transcriptase. The resulting cDNAs were PCR-amplified to about 20–30 ng µl^−1^ using a high fidelity DNA polymerase. The primers used for PCR amplification were designed for TruSeq sequencing according to the instructions of Illumina. The following adapters sequences flank the cDNA inserts (the NNNNNN indicates the barcode sequence used for multiplexing): 5′-end: 5′-AAT GAT ACG GCG ACC ACC GAG ATC TAC ACT CTT TCC CTA CAC GAC GCT CTT CCG ATC T-3′ and 3′-end: 5′-CAA GCA GAA GAC GGC ATA CGA GAT-NNN NNN-GTG ACT GGA GTT CAG ACG TGT GCT CTT CCG ATC TTT TTT TTT TTT TTT TTT TTT TTT T-3′. The cDNA libraries were purified using the Agencourt AMPure XP kit (Beckman Coulter Genomics), analyzed by capillary electrophoresis and finally sequenced by an Illumina GAIIx machine.

The sequences of the obtained sequencing reads were quality trimmed by the program *fastq_quality_trimmer* from the FASTX program suite with a cut-off phred score of 20. Poly(A) tail sequences were clipped from the 3′ end of the sequences, the resulting sequences were filtered by length and sequences short than 12 nt were discarded. The remaining reads were aligned to the reference genome sequences (accession numbers: CP000143.1, CP000144.1, CP000145.1, CP000146.1, CP000147.1, DQ232586.1, DQ232587.1) using the short read mapper *segemehl* [70]. Based on these read mapping, coverage plots which represent the number of mapped reads per nucleotide were created. Those were visualized and examined in the Integrated Genome Browser [71].

## Supporting Information

Dataset S1 Microarray and SILAC data for the singlet oxygen stress response. Relative changes (log_2_ ratios) after singlet oxygen stress were calculated for the transcriptome (Total), translatome (Poly), and proteome (SILAC). The dataset contains columns with following information from left to right: Gene Annotation (RSP), Gene Name, Description, Total 7 min log_2_, Total 7 min p-value, Total 45 min log_2_, Total 45 min p-value, Total 90 min log_2_, Total 90 min p-value, Poly 90 min log_2_, Poly 90 min p-value, SILAC 90 min log_2_, SILAC 90 min p-value, RpoE regulon, ^1^O_2_ affected, RpoH2 promoter, 2D RpoH2, 2D RpoH1-2, ChIP RpoH1-2, IMG Systematic, Probe Identifier.
(XLSX)

Dataset S2 Results of SILAC experiments. The first sheet contains SILAC H/L-ratios of identified proteins from the heavy standard. The second sheet shows the SILAC results of the stressed and unstressed *R. sphaeroides* samples.
(XLSX)

Figure S1 Scatter-plots for biological replicates of microarray experiments. Correlations between biological replicates 1 and 2 of microarray experiments were calculated as Pearson's *r* for log_2_ ratios and visualized as scatter-plots. “Total RNA” refers to transcriptome and “Poly RNA” to translatome experiments. (A) 7 min versus 0 min transcriptome, (B) 45 min versus 0 min transcriptome, (C) 90 min versus 0 min transcriptome, and (D) 90 min versus 0 min translatome. Histograms at the top and right-hand side display log_2_ ratio distributions of individual replicates.
(PDF)

Figure S2 Supporting data for transcriptionally up-regulated genes. Changes in the transcriptome after singlet oxygen stress were calculated as log_2_ ratios relative to control conditions (Cy3/Cy5). (A) Overlap (number of shared features) between two alternative RpoH2 regulons. Regulon predictions of Nuss et al. (2009, 2010) were based on genome-wide promoter searches [28], [29], while Dufour et al. (2012) used expression profiles and ChIP-chip experiments for predictions [35]. All corresponding mRNAs, exhibiting log_2_ ratios ≥0.8 at one of the experimental time-points (7, 45, 90 min) in this study, were considered and depicted in a Venn diagram. (B) Expression kinetics of the two alternative RpoH2 regulons, as described in (B). (C) Venn diagram depicting the overlap (number of shared features) of up-regulated mRNAs (log_2_ ratio ≥0.8) between 7, 45, and 90 min samples. (D) Heat-map for sRNAs that were up-regulated (log_2_ ratio/FC ≥0.8) during singlet oxygen stress. Hierarchical clustering was performed using MeV (Multi Experiment Viewer version 4.7.4) from the TM4 Microarray Software Suite.
(PDF)

Figure S3 Evaluation of the bacterial SILAC standard. The bacterial SILAC standard was prepared from heavy labeled *R. sphaeroides* cells which were grown under semi-aerobic, aerobic, and singlet oxygen stress conditions. An average Lys6-incorporation of 96% was detected for the heavy standard as shown in the histogram. Incorporation rates are plotted against relative numbers of identified proteins.
(PDF)

Figure S4 Scatter-plots for biological replicates of SILAC experiments. Pairwise comparison between biological replicates of SILAC experiments (insol and ingel digest) reveals high Pearson correlation *r* ranging between 0.86 and 0.94. Scatter-plots represent SILAC ratios (log_2_) of each experiment.
(PDF)

Figure S5 Enrichment of polysomes by sucrose gradients. Polysomes were enriched by sucrose density gradient centrifugation of crude *R. sphaeroides* extracts. (A) Picture of a sucrose gradient (0.9–1.8 M) after ultra-centrifugation (200.000*g*) of crude extracts. The gradient was divided into nine fractions, which were collected for further analysis. The pellet was enriched for polysomes (polysome fraction) and subsequently used for translatome analysis in this study. Membrane fractions were visible as two colored rings. The nine sucrose gradient fractions were analyzed on ethidium-bromide-stained urea-polyacrylamide-gels (B) and corresponding RNA concentrations were determined by spectroscopy at 260 nm (C). tRNAs were found in fractions 3–5, while ribosomal RNAs (5.8S and 5S rRNAs) were present in fractions 7–9. (D) Urea-polyacrylamide-gel loaded with RNA from polysome fractions and total RNA was stained with ethidium bromide. RNA samples from polysome fractions before and after singlet oxygen stress (Poly 0 min and 90 min ^1^O_2_) were enriched for ribosomal RNAs (16S, 14S, 5.8S, and 5S rRNA) and depleted for tRNAs when compared to total RNA. Please note that in *Rhodobacter* species and related alpha-proteobacteria 23S rRNA is fragmented into an additional 16S-like rRNA (1.5 kb), 14S rRNA (1.1 kb), and 5.8S-like rRNA.
(PDF)

Figure S6 Expression patterns within stress-inducible operons. Normalized fluorescence values, reflecting RNA expression levels, are depicted for “Total RNA” at 0 min (light grey bars) and 90 min (black bars) as well as for “Poly RNA” at 0 min (orange bars) and 90 min (red bars). Orientation of genes within operons is depicted from the left to the right, irrespective of their location in the genome, with the leftmost gene representing the first gene in the operon. Gene numbers refer to corresponding RSP-numbers. Compare (A–H) to Figure 6 for further details.
(PDF)

Table S1 Biological function of genes within transcriptional expression clusters.
(PDF)

Table S2 Biological function of genes within RpoE and RpoH2 regulons.
(PDF)

Table S3 Biological function of regulated proteins identified by SILAC-based MS.
(PDF)

Table S4 Putative new open reading frames (ORFs) identified by SILAC-based MS.
(PDF)

Table S5 New singlet oxygen-responsive genes found in this study.
(PDF)
