# Supplementary figures and images for: Integrative “Omics”-Approach Discovers Dynamic and Regulatory Features of Bacterial Stress Responses
Source: PLoS Genet. 2013 Jun 20;9(6):e1003576. doi: 10.1371/journal.pgen.1003576 (PMC3688512; doi:10.1371/journal.pgen.1003576)

# Supporting Information Figure S1

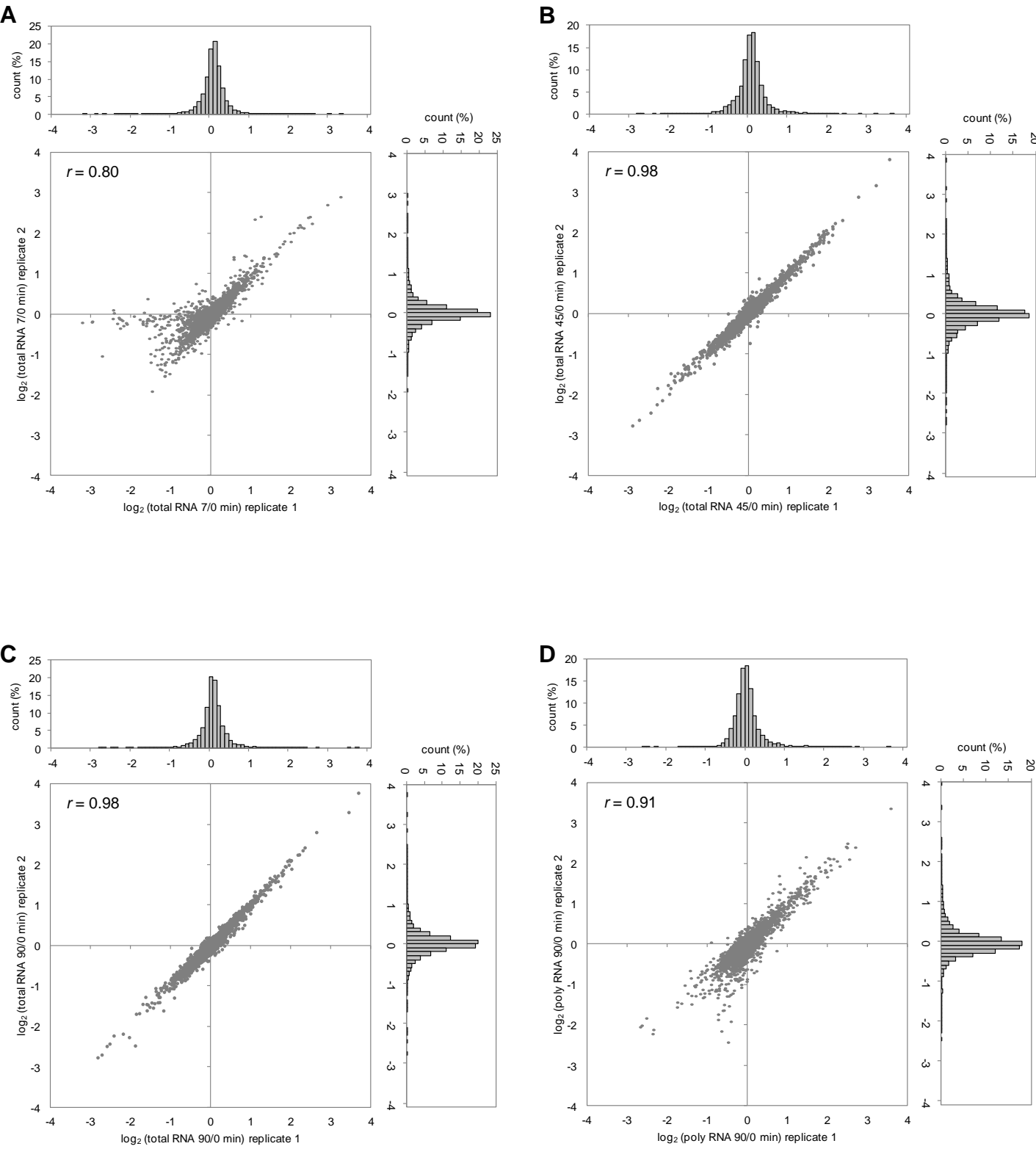

Supplement: Figure S1 — Scatter-plots for biological replicates of microarray experiments. Correlations between biological replicates 1 and 2 of microarray experiments were calculated as Pearson's r for log2 ratios and visualized as scatter-plots. “Total RNA” refers to transcriptome and “Poly RNA” to translatome experiments. (A) 7 min versus 0 min transcriptome, (B) 45 min versus 0 min transcriptome, (C) 90 min versus 0 min transcriptome, and (D) 90 min versus 0 min translatome. Histograms at the top and right-hand side display log2 ratio distributions of individual replicates. (PDF) [file pgen.1003576.s003.pdf]

# Supporting Information Figure S2

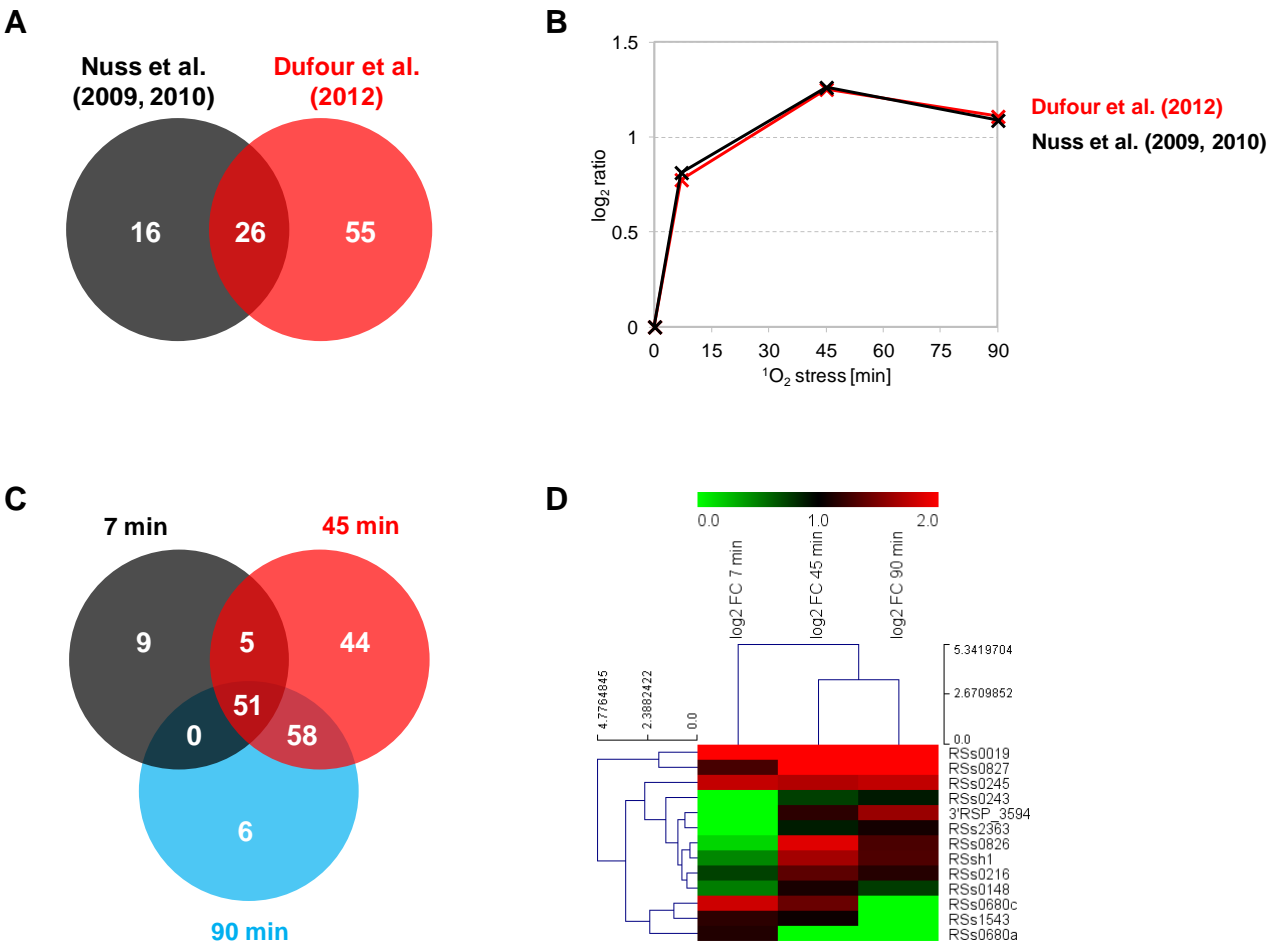

Supplement: Figure S2 — Supporting data for transcriptionally up-regulated genes. Changes in the transcriptome after singlet oxygen stress were calculated as log2 ratios relative to control conditions (Cy3/Cy5). (A) Overlap (number of shared features) between two alternative RpoH2 regulons. Regulon predictions of Nuss et al. (2009, 2010) were based on genome-wide promoter searches [28], [29], while Dufour et al. (2012) used expression profiles and ChIP-chip experiments for predictions [35]. All corresponding mRNAs, exhibiting log2 ratios ≥0.8 at one of the experimental time-points (7, 45, 90 min) in this study, were considered and depicted in a Venn diagram. (B) Expression kinetics of the two alternative RpoH2 regulons, as described in (B). (C) Venn diagram depicting the overlap (number of shared features) of up-regulated mRNAs (log2 ratio ≥0.8) between 7, 45, and 90 min samples. (D) Heat-map for sRNAs that were up-regulated (log2 ratio/FC ≥0.8) during singlet oxygen stress. Hierarchical clustering was performed using MeV (Multi Experiment Viewer version 4.7.4) from the TM4 Microarray Software Suite. (PDF) [file pgen.1003576.s004.pdf]

Supporting Information Figure S3

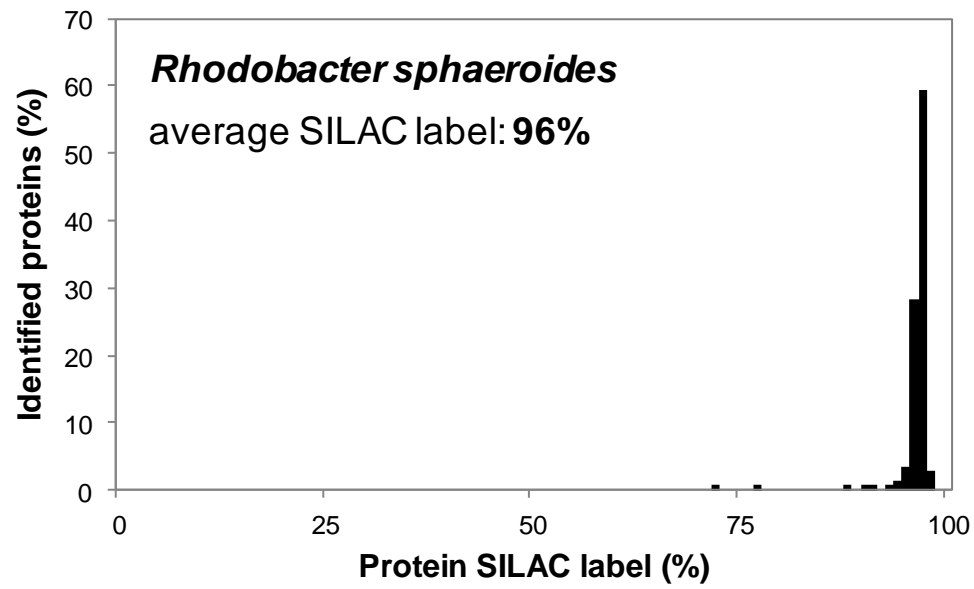

Supplement: Figure S3 — Evaluation of the bacterial SILAC standard. The bacterial SILAC standard was prepared from heavy labeled R. sphaeroides cells which were grown under semi-aerobic, aerobic, and singlet oxygen stress conditions. An average Lys6-incorporation of 96% was detected for the heavy standard as shown in the histogram. Incorporation rates are plotted against relative numbers of identified proteins. (PDF) [file pgen.1003576.s005.pdf]

# Supporting Information Figure S5

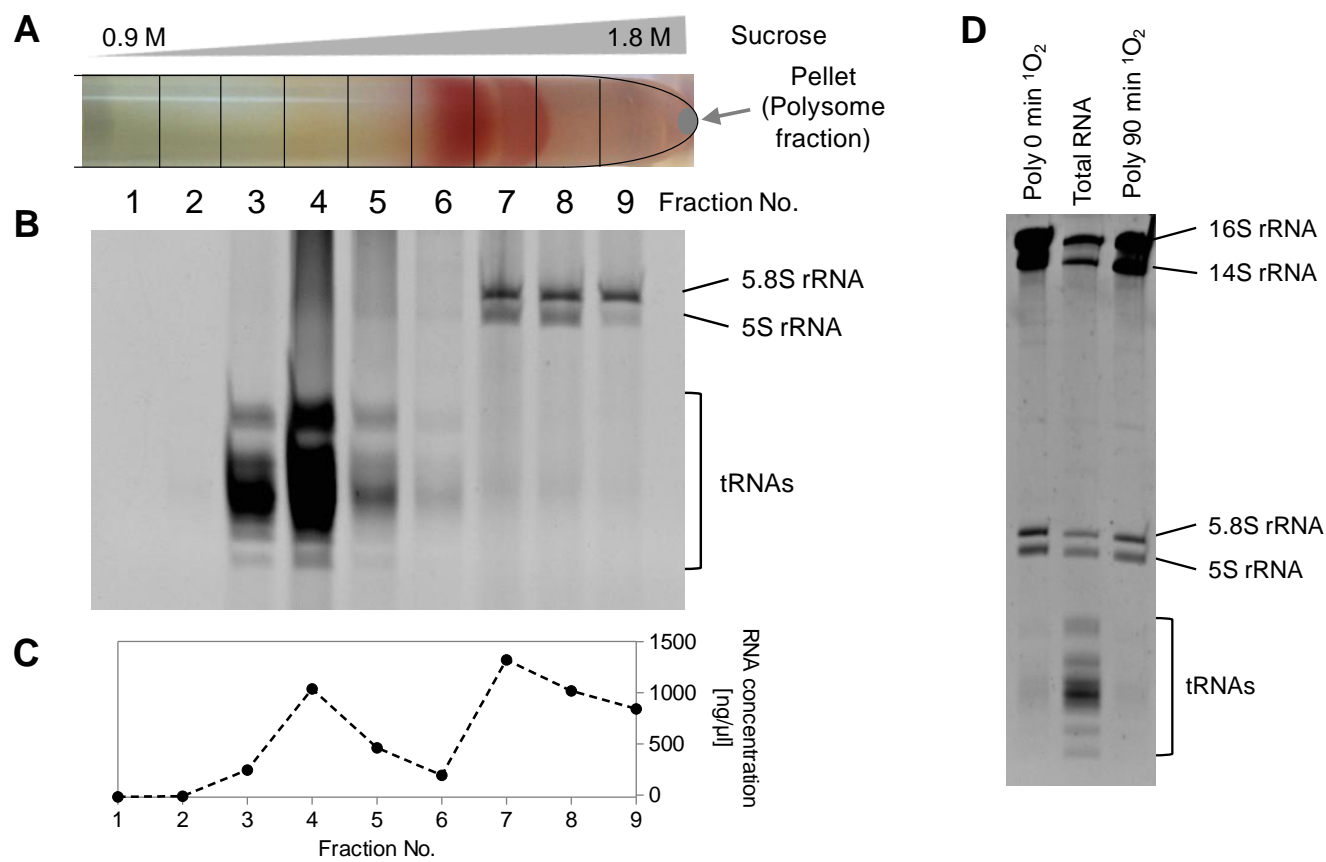

Supplement: Figure S5 — Enrichment of polysomes by sucrose gradients. Polysomes were enriched by sucrose density gradient centrifugation of crude R. sphaeroides extracts. (A) Picture of a sucrose gradient (0.9–1.8 M) after ultra-centrifugation (200.000g) of crude extracts. The gradient was divided into nine fractions, which were collected for further analysis. The pellet was enriched for polysomes (polysome fraction) and subsequently used for translatome analysis in this study. Membrane fractions were visible as two colored rings. The nine sucrose gradient fractions were analyzed on ethidium-bromide-stained urea-polyacrylamide-gels (B) and corresponding RNA concentrations were determined by spectroscopy at 260 nm (C). tRNAs were found in fractions 3–5, while ribosomal RNAs (5.8S and 5S rRNAs) were present in fractions 7–9. (D) Urea-polyacrylamide-gel loaded with RNA from polysome fractions and total RNA was stained with ethidium bromide. RNA samples from polysome fractions before and after singlet oxygen stress (Poly 0 min and 90 min 1O2) were enriched for ribosomal RNAs (16S, 14S, 5.8S, and 5S rRNA) and depleted for tRNAs when compared to total RNA. Please note that in Rhodobacter species and related alpha-proteobacteria 23S rRNA is fragmented into an additional 16S-like rRNA (1.5 kb), 14S rRNA (1.1 kb), and 5.8S-like rRNA. (PDF) [file pgen.1003576.s007.pdf]

# Supporting Information Figure S6

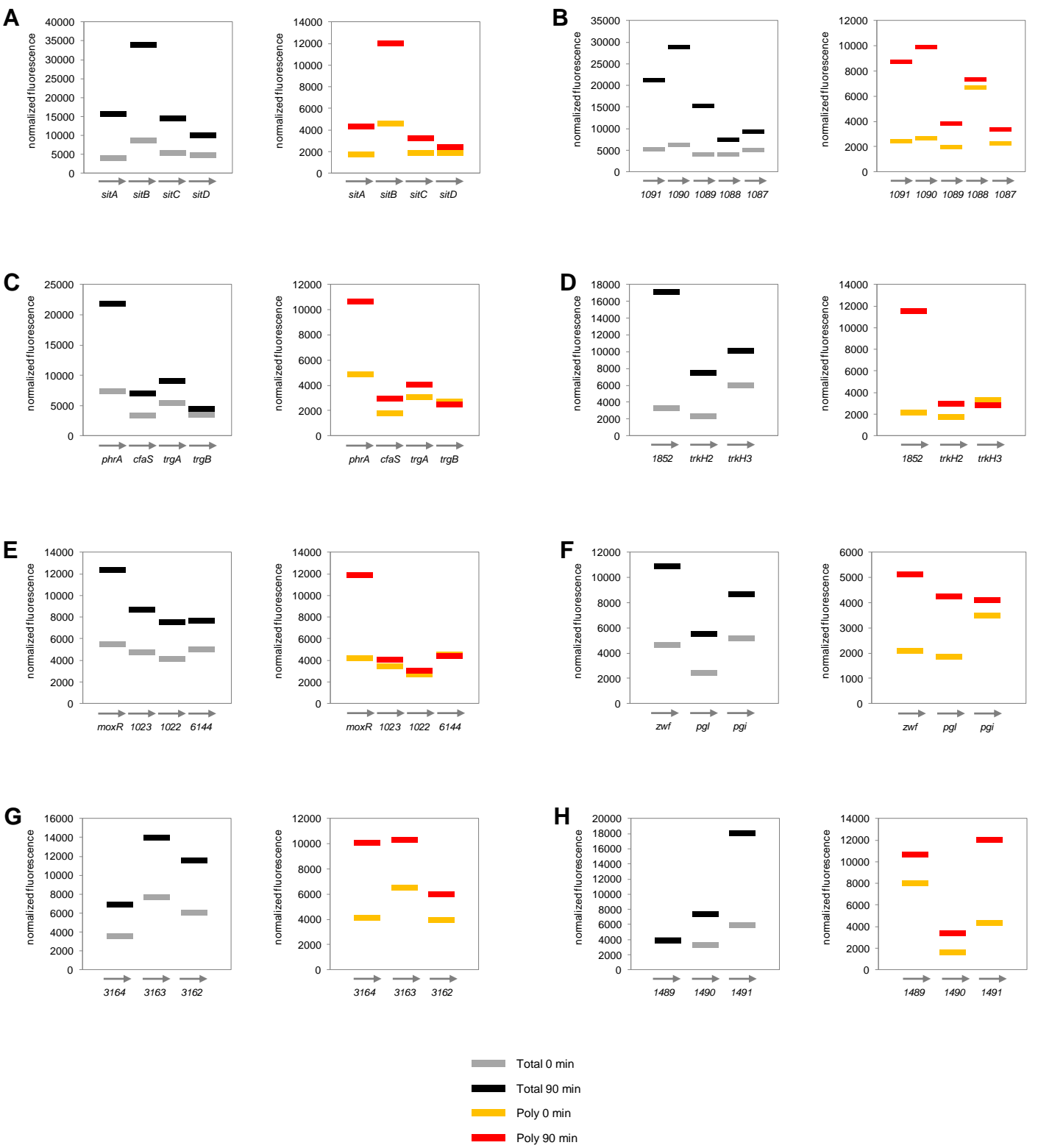

Supplement: Figure S6 — Expression patterns within stress-inducible operons. Normalized fluorescence values, reflecting RNA expression levels, are depicted for “Total RNA” at 0 min (light grey bars) and 90 min (black bars) as well as for “Poly RNA” at 0 min (orange bars) and 90 min (red bars). Orientation of genes within operons is depicted from the left to the right, irrespective of their location in the genome, with the leftmost gene representing the first gene in the operon. Gene numbers refer to corresponding RSP-numbers. Compare (A–H) to Figure 6 for further details. (PDF) [file pgen.1003576.s008.pdf]
