# Supplementary material for: Integrative “Omics”-Approach Discovers Dynamic and Regulatory Features of Bacterial Stress Responses
Source: PLoS Genet. 2013 Jun 20;9(6):e1003576. doi: 10.1371/journal.pgen.1003576 (PMC3688512; doi:10.1371/journal.pgen.1003576)

Supporting Information Figure S4

Biological replicates from Ingel-digest

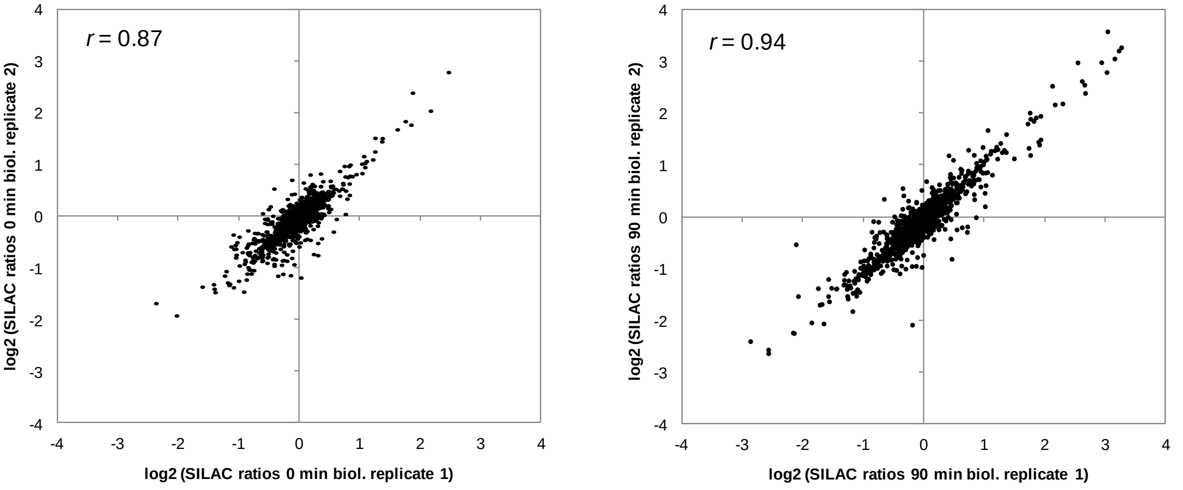

Biological replicates from Insol-digest

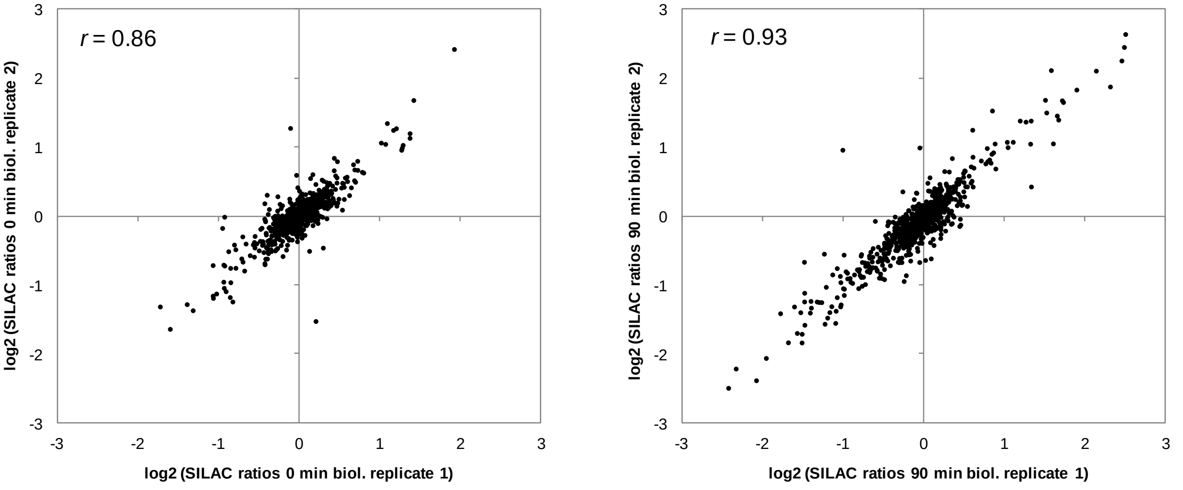

Supplement: Figure S4 — Scatter-plots for biological replicates of SILAC experiments. Pairwise comparison between biological replicates of SILAC experiments (insol and ingel digest) reveals high Pearson correlation r ranging between 0.86 and 0.94. Scatter-plots represent SILAC ratios (log2) of each experiment. (PDF) [file pgen.1003576.s006.pdf]
