# Supplementary material for: Integrative “Omics”-Approach Discovers Dynamic and Regulatory Features of Bacterial Stress Responses
Source: PLoS Genet. 2013 Jun 20;9(6):e1003576. doi: 10.1371/journal.pgen.1003576 (PMC3688512; doi:10.1371/journal.pgen.1003576)
Supplement: Table S1 — Biological function of genes within transcriptional expression clusters. (PDF) [file pgen.1003576.s009.pdf]

Table S1. Biological function of genes within transcriptional expression clusters.

Category/ Gene No.	Gene Name	7 min	log_2 ratio 45 min	90 min	Gene description/function
**Cluster 1**					
**Regulators**					
RSP_0601	*rpoH2*	1.99	2.13	2.24	RNA polymerase sigma factor
RSP_0847	*ompR*	2.45	1.48	0.72	two component transcriptional regulator, winged helix family
RSP_1092	*rpoE*	2.23	1.95	2.05	RNA polymerase sigma-70 factor
RSP_1093	*chrR*	2.05	2.20	2.33	Anti-sigma factor ChrR
RSP_2410	*rpoH1*	1.55	1.85	1.43	RNA polymerase sigma factor
**Stress defense**					
RSP_0799	*gloB*	1.35	2.11	1.84	Putative hydroxyacylglutathione hydrolase: glyoxalase II
RSP_1591	*gst*	1.55	1.92	1.92	Predicted Glutathione S-transferase
RSP_2143	*phrA*	1.58	1.46	1.58	DNA photolyase, Cryptochrome 1 apoprotein (Blue light photoreceptor)
RSP_2389		0.99	1.69	1.43	putative glutathione peroxidase
RSP_2617	*msrB*	0.88	1.83	1.50	Peptide methionine sulfoxide reductase
RSP_3212	*qxtA*	1.20	2.02	1.60	Quinol oxidase subunit I QxtA
**Iron metabolism**					
RSP_0904	*sitA*	0.80	1.81	1.96	ABC Mn+2/Fe+2 transporter, periplasmic substrate-binding protein SitA
RSP_0905	*sitB*	0.80	1.72	1.98	ABC Mn+2/Fe+2 transporter, ATPase subunit SitB
RSP_1546	*bfr*	1.14	1.79	1.60	Bacterioferritin
**Others**					
RSP_0600		2.21	1.89	2.06	hypothetical protein
RSP_1089		1.91	1.85	1.91	sugar/cation symporter, GPH family
RSP_1090		2.40	1.94	2.21	Putative cyclopropane/cyclopropene fatty acid synthesis protein
RSP_1091		2.16	1.97	2.00	Putative cyclopropane/cyclopropene fatty acid synthesis protein, flavin amine oxidase
RSP_1409		2.81	4.47	4.58	Beta-Ig-H3/Fasciclin
RSP_1491		1.09	1.70	1.61	hypothetical protein
RSP_1760		1.06	1.81	1.56	hypothetical protein
RSP_1852	*folE2*	2.22	2.33	2.39	GTP cyclohydrolase
RSP_1853	*trkH2*	1.24	1.65	1.66	potassium uptake transporter, transmembrane component, TrkH
RSP_2314		0.68	1.75	1.66	oxidoreductase, Chromogranin/secretogranin
RSP_2718		1.69	2.82	2.72	possible outer membrane protein
RSP_3075		0.95	1.65	1.52	hypothetical protein
RSP_3076		1.06	1.85	1.79	hypothetical protein
RSP_3537		1.13	1.76	1.59	alcohol dehydrogenase, zinc-containing
RSP_3866		1.83	2.00	1.79	conserved hypothetical protein
RSP_4209		1.42	1.59	1.41	Acyltransferase 3 family
RSP_6229		1.15	1.56	1.44	hypothetical protein
**Cluster 2A**					
**Stress defense**					
RSP_0392	*gloA*	1.00	1.32	1.18	probable lactoylglutathione lyase
RSP_2268		0.98	1.39	1.26	metallo Beta lactamase superfamily
RSP_2973		0.69	1.37	1.24	Peroxiredoxin
RSP_3272	*ggt*	0.99	1.25	1.31	Gamma-glutamyltranspeptidase
**Chaperones**					
RSP_0152		0.81	1.18	1.10	P-loop ATPase
RSP_1016	*ibpA*	0.98	1.40	0.96	small heat shock protein; molecular chaperone IbpA
RSP_1024	*moxR*	0.44	1.34	1.17	Putative MoxR family protein
RSP_2293	*clpA*	0.66	1.41	1.18	Chaperonin clpA/B
**Redox reaction**					
RSP_0351		0.89	1.27	1.02	D-threo-aldose 1-dehydrogenase
RSP_0398	*gluD*	0.47	1.56	1.46	dehydrogenase
RSP_1410	*yedY*	0.82	1.59	1.30	Sulfite oxidase subunit YedY
RSP_2734	*zwf*	0.49	1.10	1.22	glucose-6-phosphate 1-dehydrogenase
RSP_2785	*cycF*	0.40	1.78	1.71	Cytochrome c-554
**Others**					
RSP_0370		0.92	1.43	1.27	hypothetical protein
RSP_0595		0.70	1.38	1.45	hypothetical protein
RSP_0906	*sitC*	0.66	1.25	1.41	ABC Mn+2/Fe+2 transporter, inner membrane subunit SitC
RSP_1258		1.10	1.52	1.34	putative hydrolase
RSP_1421		0.91	1.42	1.13	hypothetical protein
RSP_1490		1.11	1.48	1.18	Putative ATP-dependent protease La, LON
RSP_1542		0.90	1.40	1.23	ABC transporter, ATPase subunit
RSP_1759		0.91	1.51	1.37	hypothetical protein
RSP_1895		0.79	1.38	1.10	Small-conductance mechanosensitive ion channel
RSP_2163		0.66	1.35	1.20	lytic murein transglycosylase, putative
RSP_2225		0.64	1.33	1.08	hypothetical protein
RSP_2388		0.81	1.58	1.30	hypothetical protein

RSP_2390	*acuC1*	0.96	1.22	1.17	putative Acetoin utilization protein
RSP_3089		0.65	1.23	1.06	hypothetical protein
RSP_3265		0.41	1.52	1.04	hypothetical protein
RSP_3327		0.85	1.31	1.14	possible Rhomboid family membrane protein
RSP_3351		1.23	1.23	1.16	hypothetical protein
RSP_6016		0.84	1.50	1.16	hypothetical protein
RSP_6106		0.80	1.34	1.41	hypothetical protein
RSP_6107		0.89	1.57	1.30	hypothetical protein
**Cluster 2B**					
**Stress defense**					
RSP_2144	*cfaS*	1.37	1.04	1.07	Cyclopropane-fatty-acyl-phospholipid synthase CfaS
RSP_2413	*lig2*	0.82	0.97	0.96	DNA ligase
RSP_2966	*uvrA*	0.69	0.88	0.64	excinuclease ABC subunit A
RSP_3210	*qxtB*	1.12	1.13	1.10	Quinol oxidase subunit II QxtB
**Chaperones**					
RSP_1207	*hslO*	0.84	1.01	0.67	putative Hsp33 protein
RSP_2310	*groES*	1.37	1.30	0.51	Chaperonin Cpn10 (GroES)
**Redox reaction**					
RSP_1087		0.91	0.68	0.88	Short-chain dehydrogenase/reductase family member
RSP_1411	*yedZ*	0.69	1.23	0.94	sulfite oxidase subunit YedZ
RSP_3163		0.99	0.86	0.86	Probable oxidoreductase
RSP_3164		0.73	1.15	0.97	ferredoxin like protein
**Transport process**					
RSP_2803		0.85	1.05	0.88	multidrug/cation efflux pump, RND superfamily
RSP_3273		0.98	1.08	0.91	ABC multidrug/carbohydrate efflux transporter, inner membrane subunit
RSP_3274		0.98	0.86	0.78	ABC multidrug/carbohydrate efflux transporter, ATPase subunit
RSP_3603		0.94	1.18	0.91	Possible ABC efflux tranporter, fused inner membrane domains
**Porphyrin metabolism**					
RSP_0602	*cobP*	1.24	1.19	1.01	Possible adenosyl cobinamide kinase/ cobinamide phosphate guanylyltransferase
RSP_1197	*hemH*	0.82	1.15	0.88	ferrochelatase
**Others**					
RSP_0011		0.73	0.93	0.82	Predicted hydrolase or acyltransferase (alpha/beta hydrolase)
RSP_0013		0.98	0.91	0.82	hypothetical protein
RSP_0472		0.67	1.03	1.01	hypothetical protein
RSP_0473		0.78	0.85	0.73	phospholipase-D family protein
RSP_0569		0.71	1.05	0.79	hypothetical protein
RSP_0570		0.70	0.95	0.74	hypothetical protein
RSP_1025		1.17	0.91	1.00	hypothetical protein
RSP_1088		1.11	0.81	0.88	hypothetical protein
RSP_1497		0.78	0.88	0.35	Putative outer membrane lipoprotein carrier protein
RSP_1540		0.66	1.16	1.00	Predicted secreted hydrolase
RSP_1692	*pduO*	0.66	1.18	1.03	hypothetical protein
RSP_1840		0.86	0.97	0.96	hypothetical protein
RSP_1985		0.86	0.96	0.97	hypothetical protein
RSP_2391	*ligT*	0.81	0.89	0.83	2'-5' RNA ligase
RSP_2414		0.64	1.05	1.04	putative mRNA 3-end processing factor
RSP_2421		0.67	1.13	1.00	hypothetical protein
RSP_2569	*sqdB*	0.68	0.85	0.78	sulfolipid (UDP-sulfoquinovose) biosynthesis protein
RSP_2735	*pgl*	0.59	0.99	1.18	6-phosphogluconolactonase
RSP_3162		1.16	0.96	0.94	hypothetical protein
RSP_6176		0.76	0.92	0.73	putative tail fiber assembly protein
**Cluster 2C**					
**Stress defense**					
RSP_0559	*msrA*	0.32	0.82	0.65	Peptide methionine sulfoxide reductase
RSP_0725		0.39	1.08	0.88	Thioredoxin, thioldisulfide interchange protein
RSP_0899		0.39	1.04	0.76	Thiol peroxidase
RSP_1397	*gst*	0.40	0.91	0.79	Glutathione S-transferase
RSP_2083	*uvrB*	0.60	0.97	0.78	excinuclease ABC subunit B
RSP_2294	*gloB*	0.46	0.86	0.75	putative hydroxyacylglutathione hydrolase (glyoxalase II) (GLX II) protein
RSP_2693	*sodB*	0.20	1.19	1.03	Superoxide dismutase, Fe-Mn family
RSP_3077	*cryB*	0.52	1.11	1.00	Cryptochrome (deoxyribodipyrimidine photolyase-related protein)
RSP_3423	*xthA1*	0.62	0.81	0.72	Probable exodeoxyribonuclease III
**Proteases**					
RSP_0355	*degP*	0.45	0.87	0.70	possible serine protease
RSP_0356	*hflC*	0.67	0.84	0.53	HflC protein; K04087 membrane protease subunit HflC
RSP_0665	*ftsH*	0.42	0.99	0.76	membrane protease FtsH catalytic subunit
RSP_0686	*clpS*	0.36	0.82	0.76	ATP-dependent Clp protease adaptor protein clpS
RSP_1076	*sohB*	0.55	1.18	0.80	Peptidase family S49
RSP_1096		0.56	1.27	0.93	putative zinc protease
RSP_1097		0.41	1.10	0.66	putative zinc protease

**Redox reaction**					
RSP_0423		0.44	0.80	0.58	Aldo/keto reductase
RSP_1545		0.64	0.95	0.73	Probable thiol oxidoreductase with 2 cytochrome c heme-binding sites
RSP_2199		0.59	0.90	0.48	putative acyl-CoA dehydrogenase
RSP_2784	*cycG*	0.40	1.11	1.07	Diheme class I cytochrome c
RSP_3183		0.50	0.90	0.97	3-hydroxyacyl-CoA dehydrogenase type II
**Transport process**					
RSP_0908	*sitD*	0.45	0.92	1.06	ABC Mn+2/Fe+2 transporter, inner membrane subunit SitD
RSP_1204		0.62	0.94	0.66	ABC multidrug efflux pump, fused ATPase and inner membrane subunits
RSP_2373		0.24	0.87	0.76	Putative Transporter, Major facilitator superfamily (MFS)
RSP_2608	*corA*	0.60	0.88	0.62	magnesium/cobalt transport protein, MIT family
RSP_2802		0.54	1.21	0.95	multidrug/cation efflux pump, membrane fusion protein subunit
RSP_2854		0.64	0.94	0.77	Cation/multidrug efflux pump, Membrane fusion protein (MFP) family
**Carbohydrate metabolism**					
RSP_1415		0.16	1.07	0.98	putative polysaccharide deacetylase
RSP_2115	*envA*	0.34	1.07	1.18	putative UDP-3-O-acyl N-acetylglucosamine deacetylase
RSP_2550	*exoK*	0.34	1.02	0.74	endo-beta-1,3-1,4-glycanase protein
RSP_2645	*eda*	0.07	0.53	0.88	KDPG/KHG bifunctional aldolase
RSP_4045	*fbaB*	0.16	0.84	0.50	fructose-bisphosphate aldolase
**Regulators**					
RSP_1083		0.10	0.71	0.88	two component transcriptional regulator, winged helix family
RSP_2719	*lrp*	0.24	0.86	0.73	transcriptional regulator, AsnC/Lrp family
RSP_2853		0.45	1.04	0.74	Transcriptional regulator, TetR family
**Others**					
RSP_0119		0.46	1.15	0.88	PA-phosphatase related phosphoesterase
RSP_0730		0.45	0.88	0.77	hypothetical protein
RSP_0850		0.48	0.83	1.08	hypothetical protein
RSP_0870		0.55	1.12	0.92	hypothetical protein
RSP_1000		0.27	0.91	0.80	hypothetical protein
RSP_1022		0.47	0.95	0.86	conserved hypothetical protein (possibly transmembrane)
RSP_1023		0.44	1.03	0.88	hypothetical protein
RSP_1026		0.56	0.87	0.78	hypothetical protein
RSP_1199		0.19	1.07	1.01	secreted conserved hypothetical protein
RSP_1399		0.52	0.86	0.73	N-formylglutamate amidohydrolase
RSP_1825	*tldD*	0.52	0.99	0.79	probable modulator of DNA gyrase
RSP_2202		-0.04	0.68	0.81	hypothetical protein
RSP_2218		0.40	1.09	0.89	hypothetical protein
RSP_2235		0.62	0.84	0.64	hypothetical protein
RSP_2480		0.49	1.00	0.89	hypothetical protein
RSP_2481	*cysE*	0.49	0.88	0.77	serine acetyltransferase
RSP_2625	*nifU*	0.47	0.75	0.83	NifU-related protein involved in Fe-S cluster formation
RSP_2646	*edd*	0.29	0.91	1.16	Pseudogene
RSP_2763		0.36	0.93	0.76	hypothetical protein
RSP_3138		0.57	0.93	0.79	Smp-30/Cgr1 family protein
RSP_3305	*abc1*	0.41	0.95	0.77	putative ubiquinol-cytochrome-c reductase assembly protein
RSP_3310		0.30	1.01	0.92	Pseudogene
RSP_3331		-0.07	0.69	0.86	Cupin domain containg protein
RSP_3601		0.45	1.02	0.82	Lipolytic enzyme, G-D-S-L family
RSP_3653		0.34	0.95	0.51	hypothetical protein
RSP_6022		0.07	0.93	0.63	hypothetical protein
RSP_6156		0.37	0.82	0.67	hypothetical protein
**Cluster 3**					
**Chaperones**					
RSP_1173	*dnaK*	0.93	0.81	0.23	Heat shock protein (Hsp70, Dank)
RSP_1408	*clpB*	0.84	0.45	0.27	Chaperone ClpB
RSP_1532	*hslU*	0.82	0.14	-0.11	ATP-dependent protease ATP-binding subunit
RSP_2311	*groEL*	1.58	1.09	0.41	chaperonin GroEL
**Amino acid & sulfur metabolism**					
RSP_1352	*serA*	1.15	-0.56	-0.63	D-3-phosphoglycerate dehydrogenase
RSP_1941	*cysH*	1.08	-0.36	-0.25	phosphoadenosine phosphosulfate reductase
RSP_1942	*cysI*	1.13	-0.14	0.02	Sulfite/nitrite reductase hemoprotein subunit
RSP_1944	*cysG*	1.22	0.02	0.17	Uroporphiryn-III C-methyltransferase/siroheme synthase
**Others**					
RSP_0846	*ribA*	0.97	0.55	0.71	GTP cyclohydrolase II
RSP_2661		0.90	0.29	0.43	Iron-containing alcohol dehydrogenase
RSP_1854	*trkH3*	1.07	0.57	0.74	potassium uptake transporter, transmembrane component, TrkH
