# Supplementary material for: Integrative “Omics”-Approach Discovers Dynamic and Regulatory Features of Bacterial Stress Responses
Source: PLoS Genet. 2013 Jun 20;9(6):e1003576. doi: 10.1371/journal.pgen.1003576 (PMC3688512; doi:10.1371/journal.pgen.1003576)
Supplement: Table S2 — Biological function of genes within RpoE and RpoH2 regulons. (PDF) [file pgen.1003576.s010.pdf]

Table S2. Biological function of genes within RpoE and RpoH2 regulons.

Category/ Gene No.	Gene Name	log$_2$ ratio 7 min	45 min	90 min	Gene description/function
**RpoE regulon**					
RSP_2144	*cfaS*	1.37	1.04	1.07	Cyclopropane-fatty-acyl-phospholipid synthase CfaS
RSP_1409		2.81	4.47	4.58	Beta-Ig-H3/Fasciclin
RSP_1091		2.16	1.97	2.00	Putative cyclopropane/cyclopropene fatty acid synthesis protein, flavin amine oxidase
RSP_1852	*folE2*	2.22	2.33	2.39	GTP cyclohydrolase
RSP_1090		2.40	1.94	2.21	Putative cyclopropane/cyclopropene fatty acid synthesis protein
RSP_1092	*rpoE*	2.23	1.95	2.05	RNA polymerase sigma-70 factor
RSP_1093	*chrR*	2.05	2.20	2.33	Anti-sigma factor ChrR
RSP_0601	*rpoH2*	1.99	2.13	2.24	RNA polymerase sigma factor
RSP_1089		1.91	1.85	1.91	sugar/cation symporter, GPH family
RSP_2143	*phrA*	1.58	1.46	1.58	DNA photolyase, Cryptochrome 1 apoprotein (Blue light photoreceptor)
RSP_1088		1.11	0.81	0.88	hypothetical protein
RSP_1087		0.91	0.68	0.88	Short-chain dehydrogenase/reductase family member
**RpoH2 regulon**					
RSP_0355	*degP*	0.45	0.87	0.70	possible serine protease
RSP_0392	*gloA*	1.00	1.32	1.18	probable lactoylglutathione lyase
RSP_0398	*gluD*	0.47	1.56	1.46	dehydrogenase
RSP_0423		0.44	0.80	0.58	Aldo/keto reductase
RSP_0799	*gloB*	1.35	2.11	1.84	Putative hydroxyacylglutathione hydrolase: glyoxalase II
RSP_0847	*ompR*	2.45	1.48	0.72	two component transcriptional regulator, winged helix family
RSP_0899		0.39	1.04	0.76	Thiol peroxidase
RSP_1096		0.56	1.27	0.93	putative zinc protease
RSP_1097		0.41	1.10	0.66	putative zinc protease
RSP_1173	*dnaK*	0.93	0.81	0.23	Heat shock protein (Hsp70, Dank)
RSP_1207	*hslO*	0.84	1.01	0.67	putative Hsp33 protein
RSP_1397	*gst*	0.40	0.91	0.79	Glutathione S-transferase
RSP_1410	*yedY*	0.82	1.59	1.30	Sulfite oxidase subunit YedY
RSP_1415		0.16	1.07	0.98	putative polysaccharide deacetylase
RSP_1421		0.91	1.42	1.13	hypothetical protein
RSP_1497		0.78	0.88	0.35	Putative outer membrane lipoprotein carrier protein
RSP_1532	*hslU*	0.82	0.14	-0.11	ATP-dependent protease ATP-binding subunit
RSP_1591	*gst*	1.55	1.92	1.92	Predicted Glutathione S-transferase
RSP_1825	*tldD*	0.52	0.99	0.79	probable modulator of DNA gyrase
RSP_1852	*folE2*	2.22	2.33	2.39	GTP cyclohydrolase
RSP_2268		0.98	1.39	1.26	metallo Beta lactamase superfamily
RSP_2294	*gloB*	0.46	0.86	0.75	putative hydroxyacylglutathione hydrolase (glyoxalase II) (GLX II) protein
RSP_2314		0.68	1.75	1.66	oxidoreductase, Chromogranin/secretogranin
RSP_2481	*cysE*	0.49	0.88	0.77	serine acetyltransferase
RSP_2617	*msrB*	0.88	1.83	1.50	Peptide methionine sulfoxide reductase
RSP_2645	*eda*	0.07	0.53	0.88	KDPG/KHG bifunctional aldolase
RSP_2735	*pgl*	0.59	0.99	1.18	6-phosphogluconolactonase
RSP_2784	*cycG*	0.40	1.11	1.07	Diheme class I cytochrome c
RSP_2785	*cycF*	0.40	1.78	1.71	Cytochrome c-554
RSP_2802		0.54	1.21	0.95	multidrug/cation efflux pump, membrane fusion protein subunit
RSP_2854		0.64	0.94	0.77	Cation/multidrug efflux pump, Membrane fusion protein (MFP) family
RSP_2973		0.69	1.37	1.24	Peroxiredoxin
RSP_3075		0.95	1.65	1.52	hypothetical protein
RSP_3076		1.06	1.85	1.79	hypothetical protein
RSP_3077	*cryB*	0.52	1.11	1.00	Cryptochrome (deoxyribodipyrimidine photolyase-related protein)
RSP_3162		1.16	0.96	0.94	hypothetical protein
RSP_3163		0.99	0.86	0.86	Probable oxidoreductase
RSP_3164		0.73	1.15	0.97	ferredoxin like protein
RSP_3210	*qxtB*	1.12	1.13	1.10	Quinol oxidase subunit II QxtB
RSP_3212	*qxtA*	1.20	2.02	1.60	Quinol oxidase subunit I QxtA
RSP_3272	*ggt*	0.99	1.25	1.31	Gamma-glutamyltranspeptidase
RSP_3537		1.13	1.76	1.59	alcohol dehydrogenase, zinc-containing
