# Supplementary material for: Integrative “Omics”-Approach Discovers Dynamic and Regulatory Features of Bacterial Stress Responses
Source: PLoS Genet. 2013 Jun 20;9(6):e1003576. doi: 10.1371/journal.pgen.1003576 (PMC3688512; doi:10.1371/journal.pgen.1003576)
Supplement: Table S3 — Biological function of regulated proteins identified by SILAC-based MS. (PDF) [file pgen.1003576.s011.pdf]

Table S3. Biological function of regulated proteins identified by SILAC-based MS.

Category/ Gene No.	Gene Name	log_2 ratio 90 min	p-value	Gene description/function
**Up-regulated (p<0.05)**				
**Stress defense**				
RSP_0559	*msrA*	1.39	0.00037159	Peptide methionine sulfoxide reductase
RSP_0725		1.05	0.01931110	Thioredoxin, thioldisulfide interchange protein
RSP_1024	*moxR*	1.69	0.00000391	Putative MoxR family protein
RSP_1219	*grpE*	0.97	0.02186640	chaperone protein GrpE (heat shock protein)
RSP_1397	*gst*	0.87	0.00000120	Glutathione S-transferase
RSP_1464	*dsbA*	0.91	0.00001458	putative periplasmic thiol-disulphide interchange protein (DsbA family)
RSP_2083	*uvrB*	1.10	0.00001731	excinuclease ABC subunit B
RSP_2144	*cfaS*	1.91	0.03318910	Cyclopropane-fatty-acyl-phospholipid synthase CfaS
RSP_2268		1.76	0.00000783	metallo Beta lactamase superfamily
RSP_2413	*lig2*	1.91	0.00044301	DNA ligase
RSP_2617	*msrB*	1.33	0.00000571	Peptide methionine sulfoxide reductase
RSP_2973		1.31	0.00009312	Peroxiredoxin
**Redox reaction**				
RSP_0351		2.34	0.00001489	D-threo-aldose 1-dehydrogenase
RSP_0398		1.05	0.00046586	dehydrogenase
RSP_0423		1.04	0.00003887	Aldo/keto reductase
RSP_1057	*merA1*	1.55	0.00869240	Putative mercuric reductase protein
RSP_1410	*yedY*	2.26	0.00000006	sulfite oxidase subunit YedY
RSP_1869	*ubiH*	0.91	0.01205060	2-octaprenyl-6-methoxyphenyl hydroxylase
RSP_2199		1.01	0.00009613	putative acyl-CoA dehydrogenase
RSP_2395		1.15	0.02870860	BCCP, cytochrome c peroxidase
RSP_2734	*zwf*	1.65	0.00001129	glucose-6-phosphate 1-dehydrogenase
RSP_2785	*cycF*	1.52	0.00003505	Cytochrome c-554
RSP_2805	*nemA*	0.87	0.00005148	NADH-flavin oxidoreductase
RSP_3163		1.04	0.00000163	Probable oxidoreductase
RSP_3537		1.85	0.00000096	alcohol dehydrogenase, zinc-containing
**Carbohydrate metabolism**				
RSP_1415		1.08	0.00000249	putative polysaccharide deacetylase
RSP_2196	*fadJ*	0.89	0.00000713	3-hydroxyacyl-CoA dehydrogenase
RSP_2197		0.91	0.00002879	acetyl-CoA acetyltransferase
RSP_2550	*exoK*	1.67	0.00001272	endo-beta-1,3-1,4-glycanase protein
RSP_2735	*pgl*	1.35	0.00002869	6-phosphogluconolactonase
RSP_2736	*pgi*	1.06	0.00038861	glucose-6-phosphate isomerase
**Transport process**				
RSP_1203		1.07	0.00288290	ABC multidrug efflux pump, fused ATPase and inner membrane subunits
RSP_1204		0.96	0.00318036	ABC multidrug efflux pump, fused ATPase and inner membrane subunits
RSP_1803	*ccmC*	1.10	0.04941210	ABC heme exporter, inner membrane subunit CcmC
RSP_3274		1.83	0.00000082	ABC multidrug/carbohydrate efflux transporter, ATPase subunit
RSP_3386		0.92	0.00004444	TRAP-T family transporter periplasmic binding protein
RSP_3602		1.60	0.00000173	ABC efflux transporter, ATPase subunit
**Proteases**				
RSP_0686	*clpS*	1.01	0.00002543	ATP-dependent Clp protease adaptor protein clpS
RSP_1076	*sohB*	0.85	0.00003405	Peptidase family S49
RSP_1096		1.61	0.00036976	putative zinc protease
RSP_1097		1.65	0.00001754	putative zinc protease
RSP_1490		1.51	0.00001940	Putative ATP-dependent protease La, LON
RSP_2293	*clpA*	1.25	0.00000059	Chaperonin clpA/B
**Others**				
RSP_0013		0.99	0.00004301	hypothetical protein
RSP_0472		1.53	0.00354578	hypothetical protein
RSP_0663	*fhs*	0.92	0.00069142	Formate-tetrahydrofolate ligase
RSP_0870		0.99	0.00027241	hypothetical protein
RSP_0890	*ttg2*	0.93	0.00431800	putative toluene tolerance protein
RSP_1025		1.29	0.00001200	hypothetical protein
RSP_1091		2.82	0.00000099	Putative cyclopropane/cyclopropene fatty acid synthesis protein, flavin amine oxidase
RSP_1197	*hemH*	0.94	0.00000402	ferrochelatase
RSP_1199		0.82	0.00622293	secreted conserved hypothetical protein
RSP_1280	*cbbX*	0.98	0.01541340	CbbX protein
RSP_1409		2.59	0.00101298	Beta-Ig-H3/Fasciclin
RSP_1465		0.88	0.00553074	hypothetical protein
RSP_1497	*lolA*	1.40	0.00083363	Putative outer membrane lipoprotein carrier protein
RSP_1540		1.63	0.00001676	Predicted secreted hydrolase
RSP_1546	*bfr*	2.22	0.00124273	Bacterioferritin
RSP_1760		1.21	0.00000598	hypothetical protein
RSP_1840		1.87	0.00077570	hypothetical protein

RSP_1852		3.79	0.00093011	GTP cyclohydrolase
RSP_2163		1.02	0.00055750	lytic murein transglycosylase, putative
RSP_2831	*cobO*	0.93	0.00409328	cob(I)yrinic acid a,c-diamide adenosyltransferase
RSP_3599	*phoH*	1.12	0.00068785	phosphate starvation-inducible protein PhoH and related proteins
RSP_3721		1.26	0.03749980	hypothetical protein
RSP_4124		1.02	0.00007905	hypothetical protein
RSP_6144		1.22	0.00651711	conserved hypothetical protein (possible transmembrane)
RSP_6229		1.66	0.00230051	hypothetical protein
**Down-regulated (p<0.05)**				
**Photosynthesis**				
RSP_0260	*bchZ*	-1.44	0.00119805	putative chlorophyllide reductase, BchZ subunit
RSP_0261	*bchY*	-1.60	0.00000950	Chlorophyllide reductase, BchY subunit
RSP_0262	*bchX*	-1.27	0.00000014	Chlorophyllide reductase, BchX subunit
RSP_0263	*bchC*	-0.92	0.00002143	3-hydroxyethyl bacteriochlorophyllide a dehydrogenase
RSP_0271	*crtI*	-0.82	0.00000107	phytoene dehydrogenase
RSP_0277	*bchP*	-1.22	0.00000189	Geranylgeranyl hydrogenase
RSP_0281	*bchE*	-1.48	0.00000036	Magnesium-protoporphyrin IX monomethylester oxidative cyclase, 66 kDa subunit
RSP_0286	*bchB*	-0.91	0.00023489	light-independent protochlorophyllide reductase subunit B
RSP_0287	*bchH*	-1.49	0.00042155	Magnesium-chelatase subunit H
RSP_0288	*chlL*	-0.99	0.00021979	protochlorophyllide reductase iron-sulfur ATP-binding protein
RSP_0292		-1.42	0.00105979	putative photosynthetic complex assembly protein
RSP_0293	*puhC*	-1.30	0.00002280	photosynthetic complex assembly protein
RSP_0294		-1.54	0.00001231	magnesium-protoporphyrin IX monomethyl ester cyclase
**Motility/chemotaxis**				
RSP_0044	*tlpT*	-1.14	0.00333636	putative cytoplasmic chemoreceptor, TlpT
RSP_0067		-0.89	0.02958180	Putative methyl-accepting chemotaxis protein
RSP_0069	*fliC*	-1.10	0.00318698	Flagellar filament protein
RSP_0077	*flgH*	-1.27	0.00112242	Flagellar L-ring protein
RSP_1905	*cpaF*	-1.05	0.03012460	Putative Flp pilus assembly protein ATPase CpaF
RSP_1909	*cpaC*	-1.11	0.00050138	Outer membrane general secretion pathway protein, Secretin, pilus assembly protein
RSP_1910	*cpaB*	-0.83	0.00657392	Flp pilus assembly protein CpaB
RSP_3083	*mcpH*	-1.01	0.01301620	methyl accepting chemotaxis protein
RSP_3708	*mcpJ*	-0.88	0.00037401	methyl accepting chemotaxis protein
**Others**				
RSP_0085		-1.13	0.00475473	multisensor hybrid histidine kinase
RSP_0465		-1.09	0.00011582	putative protease
RSP_0688		-2.10	0.00000455	Probable penicillin-binding protein
RSP_1188	*wcaG*	-1.00	0.00980639	Nucleotide sugar epimerase/dehydratase
RSP_1272		-0.84	0.00003624	RNA polymerase sigma factor
RSP_1274		-1.62	0.00017836	two-component response regulator
RSP_1425		-0.86	0.03295820	plasmid replication initiation protein
RSP_1468	*ccrM*	-2.14	0.00060974	Site-specific DNA-methyltransferase
RSP_1791	*zntA*	-0.85	0.01896920	heavy metal-translocating P-type ATPase
RSP_1818	*feoB*	-1.50	0.01878450	Fe2+ transport system protein B
RSP_2166	*putA*	-0.98	0.00535854	bifunctional proline dehydrogenase/pyrroline-5-carboxylate dehydrogenase
RSP_2380	*catC*	-0.87	0.02405100	Catalase
RSP_2446	*treS*	-0.98	0.00235158	trehalose synthase
RSP_2495	*nrd*	-2.25	0.00000093	ribonucleotide-diphosphate reductase alpha subunit
RSP_2738		-1.25	0.00007486	Probable Rhodanese-related sulfurtransferase
RSP_2774		-1.33	0.00325742	peptidoglycan binding domain-containing protein
RSP_2888	*nsrR*	-1.24	0.00234503	Transcriptional regulator
RSP_3154		-0.89	0.01720220	nitrate/sulfonate/bicarbonate ABC transporter ATPase
RSP_4157		-1.07	0.01397770	Radical SAM superfamily protein
RSP_4242		-1.93	0.02229770	Hemolysin-type calcium-binding protein
RSP_4284		-1.05	0.01019410	YD repeat-containing protein
RSP_6091		-1.85	0.02617120	hypothetical protein
RSP_6111	*ureC*	-1.21	0.04497700	Urea amidohydrolase (urease) alpha subunit
