# Supplementary material for: Integrative “Omics”-Approach Discovers Dynamic and Regulatory Features of Bacterial Stress Responses
Source: PLoS Genet. 2013 Jun 20;9(6):e1003576. doi: 10.1371/journal.pgen.1003576 (PMC3688512; doi:10.1371/journal.pgen.1003576)
Supplement: Table S4 — Putative new open reading frames (ORFs) identified by SILAC-based MS. (PDF) [file pgen.1003576.s012.pdf]

Table S4. Putative new open reading frames (ORFs) identified by SILAC-based MS.

ORF ID[1]	Position[2]	Replicon	Strand	Detected aa-seq[3]	RNA-seq[4]
ID-19ORF-14558	3019891..3020211	Chr 1	Plus	AIDACETRQAAAK<br>ARSMAAAISK<br>VAMAQGRTPEEIASI	yes
ID-19ORF-3983	798262..798396	Chr 1	Plus	EEAVYVEQPAVTTEPVYTGK<br>KEEAVYVEQPAVTTEPVYTGK	yes
ID-22ORF-3245	2491636..2491836	Chr 1	Minus	AQRNPAMDAIK<br>EEIARISQA<br>HETISTSVEK<br>IKEEIARISQA	yes
ID-23ORF-6817	1704578..1704952	Chr 1	Minus	IIAAMNAGSRIK<br>TRRVEGSAYEYESD	(yes)
ID-24ORF-1183	2930079..2930258	Chr 1	Minus	TYVGYPIPSK<br>VMVRVDEK<br>VMVRVDEKTYVGYPIPSKRAA	yes
ID-24ORF-7452	1618890..1619105	Chr 1	Minus	AAPAKPRPGAPEAGPQAGGARSAAK<br>PRPGAPEAGPQAGGARSAAK<br>RIFTGRGPK	(yes)
ID-24ORF-8403	1411515..1412336	Chr 1	Minus	FQPIPIWK<br>MVSIDSWSVK<br>(NAAVPNAIRVYFRTAPFK)	no
ID-23ORF-10842	879464..879868	Chr 1	Minus	AVSAIVVPARGDWSTAAPK<br>AVSAIVVPARGDWSTAAPKK	yes
ID-24ORF-3701	2370426..2370683	Chr 1	Minus	ARAAREAGPGDAPE<br>GPAAESREDRIK	yes
ID-30ORF-1184	723678..723917	Chr 2	Minus	GETPITIAGK<br>GETPITIAGKK<br>VVETASYTK	no
ID-29ORF-1154	709835..710188	Chr 2	Minus	INQGRSISTNVDDAVK<br>KINQGRSISTNVDDAVK	yes
ID-29ORF-854	753206..753412	Chr 2	Minus	AAIQQERAINSSARA<br>DDGSIIIMK	yes
ID-33ORF-485	82294..82856	Plasmid A	Plus	AIVRTFASQVAERTPK<br>AIVRTFASQVAERTPKK<br>APRATPTPRRRQSPSSEDK<br>APRYTIRITVEAK<br>DPVPTDMPNRIAADASAAPVVAADQPEIARGETNPVGK<br>GIITRAGGSPISFRK<br>IPDSIRAAIIAVEGANPIEPAGTAISRNIAPVFAAFVRK<br>KAEYVPTDPVVYRIDIK<br>VIEEIDRIIGDMDDK	(yes)
ID-36ORF-259	66453..66806	Plasmid A	Minus	KMQDVIASYVRTMK<br>MQDVIASYVRTMK<br>RISVDEVRAIK<br>SVHIRIDPEVFRFFK	yes
ID-36ORF-125	87675..88235	Plasmid A	Minus	ATREFVRENNRFAAIVANGIQNFIGMPK<br>YEIIRQK	yes
ID-12ORF-430	39639..39851	Plasmid C	Minus	AAIIEEYRIK<br>ASAIREIIERK	(yes)
ID-15ORF-21	3552..4511	Plasmid D	Plus	DPVRVSIEIRIPIAFANK<br>IIADGMK<br>IIADGMKK<br>KVPRYTPK<br>VPRYTPK	yes
ID-13ORF-467	66469..67086	Plasmid D	Plus	QVRYEPRIIK<br>SSVTVPIRPEVRNQREIWDIVISYGSK<br>TWPGIASFVTQYTPEK	yes
ID-41ORF-21	32681..33253	Plasmid E	Minus	EYGVDVSDK<br>TNVPENNVAIVIINGSEVSTAIHRQNK	yes

[1] Identifier for ORFs according to *Rhodobacter sphaeroides* 2.4.1 ORF database.
[2] Position of ORF query sequence on particular replicon, referring to plus-strand orientation.
[3] Amino acid (aa) sequence detected by mass spectrometry.
[4] Presence of cDNA reads, potentially representing the mRNA of a new ORF, in RNA-seq data for *R. spaheroides* 2.4.1 (yes: clear cDNA read coverage, (yes): weak cDNA read coverage, no: no cDNA read coverage above background).
