# Supplementary material for: Integrative “Omics”-Approach Discovers Dynamic and Regulatory Features of Bacterial Stress Responses
Source: PLoS Genet. 2013 Jun 20;9(6):e1003576. doi: 10.1371/journal.pgen.1003576 (PMC3688512; doi:10.1371/journal.pgen.1003576)
Supplement: Table S5 — New singlet oxygen-responsive genes found in this study. (PDF) [file pgen.1003576.s013.pdf]

Table S5. New singlet oxygen-responsive genes found in this study.

			log$_2$ ratio					
Category/ Gene No.	Gene Name	Cluster/ Source[1]	Total 7 min	Total 45 min	Total 90 min	Poly 90 min	SILAC 90 min	Gene Description/function
**Stress defense**								
RSP_0725		2C	0.39	1.08	0.88	0.81	1.05	Thioredoxin, thioldisulfide interchange protein
RSP_2413	*lig2*	2B	0.82	0.97	0.96	0.23	1.91	DNA ligase
**Chaperones**								
RSP_1024	*moxR*	2A	0.44	1.34	1.17	1.50	1.69	Putative MoxR family protein
RSP_2293	*clpA*	2A	0.66	1.41	1.18	1.56	1.25	Chaperonin clpA/B
RSP_2310	*groES*	2B	1.37	1.30	0.51	2.14	0.70	Chaperonin Cpn10 (GroES)
RSP_2311	*groEL*	3	1.58	1.09	0.41	1.59		chaperonin GroEL
**Regulators**								
RSP_1997	*lexA*	Poly	-0.25	-0.02	-0.14	0.86	-0.16	LexA repressor
RSP_2719	*lrp*	2C	0.24	0.86	0.73	0.70		transcriptional regulator, AsnC/Lrp family
RSP_2853		2C	0.45	1.04	0.74	0.26	0.65	Transcriptional regulator, TetR family
RSP_3094		Poly	0.04	0.43	0.43	0.89		Putative transmembrane anti-sigma factor
RSP_3095		Poly	-0.13	0.63	0.61	1.09		RNA polymerase sigma-70 factor, ECF subfamily
**Quorum sensing**								
RSP_0123	*cerI*	Poly	-0.38	0.35	0.55	1.56	-0.31	Autoinducer synthesis protein
RSP_6213	*cerA*	Poly	-0.26	0.51	0.49	1.62		community escape response protein
**Iron metabolism**								
RSP_0905	*sitB*	1	0.80	1.72	1.98	1.39		ABC Mn+2/Fe+2 transporter, ATPase subunit
RSP_0906	*sitC*	2A	0.66	1.25	1.41	0.77		ABC Mn+2/Fe+2 transporter, inner membrane subunit
RSP_0908	*sitD*	2C	0.45	0.92	1.06	0.34		ABC Mn+2/Fe+2 transporter, inner membrane subunit
RSP_0437	*sufC*	Poly	0.49	0.39	0.56	1.52	0.44	Suf C, ATPase
RSP_0440	*sufB*	Poly	0.48	0.49	0.70	1.19	0.38	putative SufB
**Amino acid & sulfur metabolism**								
RSP_1352	*serA*	3	1.15	-0.56	-0.63	-0.52	-0.01	D-3-phosphoglycerate dehydrogenase
RSP_1941	*cysH*	3	1.08	-0.36	-0.25	-0.02	-0.67	phosphoadenosine phosphosulfate reductase
RSP_1942	*cysI*	3	1.13	-0.14	0.02	0.11	-0.21	Sulfite/nitrite reductase hemoprotein subunit
RSP_1944	*cysG*	3	1.22	0.02	0.17	0.14		Uroporphiryn-III C-methyltransferase/siroheme synthase
**Redox reaction**								
RSP_0351		2A	0.89	1.27	1.02	1.32	2.34	D-threo-aldose 1-dehydrogenase
RSP_1411	*yedZ*	2B	0.69	1.23	0.94	0.29		sulfite oxidase subunit YedZ
RSP_1545		2C	0.64	0.95	0.73	0.18		Probable thiol oxidoreductase with 2 cytochrome c heme-binding sites
RSP_2199		2C	0.59	0.90	0.48	1.03	1.01	putative acyl-CoA dehydrogenase
RSP_2395		SILAC	-0.60	-0.19	0.42	0.13	1.15	BCCP, cytochrome c peroxidase
RSP_2661		3	0.90	0.29	0.43	0.66		Iron-containing alcohol dehydrogenase
RSP_2878	*coxS*	Poly	-0.17	-1.08	0.27	0.93	-0.21	Putative carbon-monoxide dehydrogenase small chain
RSP_2948		Poly	-0.56	-0.14	0.28	1.09	0.01	indolepyruvate ferredoxin oxidoreductase
RSP_3183		2C	0.50	0.90	0.97	1.34		3-hydroxyacyl-CoA dehydrogenase type II
**Transporters**								
RSP_1203		SILAC	0.64	0.63	0.47	0.05	1.07	ABC multidrug efflux pump, fused ATPase and inner membrane subunits
RSP_1853	*trkH2*	1	1.24	1.65	1.66	0.74		potassium uptake transporter, transmembrane component, TrkH
RSP_1854	*trkH3*	3	1.07	0.57	0.74	-0.20		potassium uptake transporter, transmembrane component, TrkH
RSP_2373		2C	0.24	0.87	0.76	0.33		Putative Transporter, Major facilitator superfamily (MFS)
RSP_3273		2B	0.98	1.08	0.91	0.20		ABC multidrug/carbohydrate efflux transporter, inner membrane subunit
RSP_3386		SILAC	0.17	0.45	0.63	0.68	0.92	TRAP-T family transporter, periplasmic binding protein
RSP_3603		2B	0.94	1.18	0.91	0.36		Possible ABC efflux tranporter, fused inner membrane domains
**Carbohydrate metabolism**								
RSP_2115	*envA*	2C	0.34	1.07	1.18	1.07		putative UDP-3-O-acyl N-acetylglucosamine deacetylase
RSP_2196	*fadJ*	SILAC	0.43	0.69	0.41	0.55	0.89	enoyl-CoA hydratase / 3-hydroxyacyl-CoA dehydrogenase / 3-hydroxybutyryl-CoA epimerase
RSP_2550	*exoK*	2C	0.34	1.02	0.74	0.77	1.67	endo-beta-1,3-1,4-glycanase protein

**Others**								
RSP_0439		Poly	0.55	0.32	0.44	1.04		hypothetical protein
RSP_0483		Poly	0.14	0.45	0.35	0.81		hypothetical protein
RSP_0569		2B	0.71	1.05	0.79	0.44		hypothetical protein
RSP_0600		1	2.21	1.89	2.06	1.18		hypothetical protein
RSP_0730		2C	0.45	0.88	0.77	0.06		hypothetical protein
RSP_0846	*ribA*	3	0.97	0.55	0.71	0.62		GTP cyclohydrolase II
RSP_0850		2C	0.48	0.83	1.08	1.36		hypothetical protein
RSP_1000		2C	0.27	0.91	0.80	0.36		hypothetical protein
RSP_1022		2C	0.47	0.95	0.86	0.20		conserved hypothetical protein (possibly transmembrane)
RSP_1023		2C	0.44	1.03	0.88	0.22		hypothetical protein
RSP_1027		Poly	0.21	0.47	0.25	0.85		hypothetical protein
RSP_1045	*trmD*	Poly	0.05	-0.26	-0.44	1.07		tRNA (guanine-N1-)-methyltransferase
RSP_1157	*PSrp1*	Poly	-0.56	0.12	0.47	0.80	-0.02	Ribosomal subunit interface protein Y
RSP_1199		2C	0.19	1.07	1.01	1.29	0.82	secreted conserved hypothetical protein
RSP_1280	*cbbX*	SILAC	0.23	0.48	0.38	-0.23	0.98	CbbX protein
RSP_1383		Poly	-0.24	0.21	0.12	0.85	0.28	hypothetical protein
RSP_1399		2C	0.52	0.86	0.73	0.31		N-formylglutamate amidohydrolase
RSP_1465		SILAC	0.22	0.68	0.58	0.52	0.88	hypothetical protein
RSP_1540		2B	0.66	1.16	1.00	0.42	1.63	Predicted secreted hydrolase
RSP_1759		2A	0.91	1.51	1.37	0.84		hypothetical protein
RSP_2202		2C	-0.04	0.68	0.81	0.21		hypothetical protein
RSP_2235		2C	0.62	0.84	0.64	0.17		hypothetical protein
RSP_2390	*acuC1*	2A	0.96	1.22	1.17	0.03		putative Acetoin utilization protein
RSP_2391	*ligT*	2B	0.81	0.89	0.83	0.16		2'-5' RNA ligase
RSP_2480		2C	0.49	1.00	0.89	0.98		hypothetical protein
RSP_2585		Poly	-0.65	-1.02	0.00	1.05	0.00	hypothetical protein
RSP_2613	*plsX*	Poly	0.15	0.27	0.14	0.80	-0.02	fatty acid/phospholipid synthesis protein
RSP_2624		Poly	0.10	0.58	0.37	1.08		hypothetical protein
RSP_3073		Poly	0.21	0.20	0.35	0.86		ErfK/YbiS/YcfS/YnhG family protein
RSP_3092		Poly	-0.29	0.67	0.70	1.45		hypothetical protein
RSP_3093		Poly	-0.35	0.57	0.56	0.88		Predicted integral membrane protein
RSP_3265		2A	0.41	1.52	1.04	0.26		hypothetical protein
RSP_3331		2C	-0.07	0.69	0.86	-0.25		Cupin domain containg protein
RSP_3351		2A	1.23	1.23	1.16	1.56		hypothetical protein
RSP_3509	*expE1*	Poly	-0.04	0.29	0.50	1.56		Hemolysin-type calcium-binding region, RTX
RSP_3653		2C	0.34	0.95	0.51	0.36		hypothetical protein
RSP_3866		1	1.83	2.00	1.79	2.51		conserved hypothetical protein
RSP_4124		SILAC	0.63	0.31	0.00	0.11	1.02	hypothetical protein
RSP_4254	*rnk*	Poly	0.67	0.55	0.32	1.27		putative regulator of nucleoside diphosphate kinase protein
RSP_6016		2A	0.84	1.50	1.16	1.03		hypothetical protein
RSP_6022		2C	0.07	0.93	0.63	0.69		hypothetical protein
RSP_6060		Poly	0.02	0.61	0.62	0.82		hypothetical protein
RSP_6106		2A	0.80	1.34	1.41	0.61		hypothetical protein
RSP_6107		2A	0.89	1.57	1.30	1.67	0.52	hypothetical protein
RSP_6144		SILAC	0.25	0.73	0.62	-0.04	1.22	conserved hypothetical protein (possible transmembrane)
RSP_6156		2C	0.37	0.82	0.67	0.89		hypothetical protein
RSP_6176		2B	0.76	0.92	0.73	1.24		putative tail fiber assembly protein
RSP_6229		1	1.15	1.56	1.44	0.32	1.66	hypothetical protein
RSP_6230		Poly	0.15	0.58	0.49	1.10		hypothetical protein
RSP_0602	*cobP*	2B	1.24	1.19	1.01	0.34	0.00	Possible adenosyl cobinamide kinase/ cobinamide phosphate guanylyltransferase
RSP_0356	*hflC*	2C	0.67	0.84	0.53	0.75	0.60	HflC protein; membrane protease subunit HflC

[1] Newly identified genes originating from cluster analysis (compare Fig. 2 and Table S1), from polysomal RNA analysis (Poly), or from SILAC-based proteomics (SILAC).
